# *Eubacterium limosum* modulates tumor microenvironments and produces antitumor metabolites active against colorectal cancer

**DOI:** 10.1093/ismejo/wraf130

**Published:** 2025-06-26

**Authors:** Yao Lu, Ruiting Lan, Qianhua Fan, Huijing Tang, Dalong Hu, Shuwei Zhang, Xiaoying Lin, Ruoshi Wang, Ruiqing Zhao, Hui Sun, Liyun Liu, Jianguo Xu

**Affiliations:** National Key Laboratory of Intelligent Tracking and Forecasting for Infectious Diseases, National Institute for Communicable Disease Control and Prevention, Chinese Center for Disease Control and Prevention, Beijing 102206, China; School of Biotechnology and Biomolecular Sciences, University of New South Wales, Sydney, NSW 2052, Australia; National Key Laboratory of Intelligent Tracking and Forecasting for Infectious Diseases, National Institute for Communicable Disease Control and Prevention, Chinese Center for Disease Control and Prevention, Beijing 102206, China; Department of Epidemiology, Center for Global Health, School of Public Health, Nanjing Medical University, Nanjing, Jiangsu 211166, China; National Key Laboratory of Intelligent Tracking and Forecasting for Infectious Diseases, National Institute for Communicable Disease Control and Prevention, Chinese Center for Disease Control and Prevention, Beijing 102206, China; Department of Epidemiology, Center for Global Health, School of Public Health, Nanjing Medical University, Nanjing, Jiangsu 211166, China; School of Biotechnology and Biomolecular Sciences, University of New South Wales, Sydney, NSW 2052, Australia; National Key Laboratory of Intelligent Tracking and Forecasting for Infectious Diseases, National Institute for Communicable Disease Control and Prevention, Chinese Center for Disease Control and Prevention, Beijing 102206, China; Department of Epidemiology, Center for Global Health, School of Public Health, Nanjing Medical University, Nanjing, Jiangsu 211166, China; National Key Laboratory of Intelligent Tracking and Forecasting for Infectious Diseases, National Institute for Communicable Disease Control and Prevention, Chinese Center for Disease Control and Prevention, Beijing 102206, China; Department of Epidemiology, Center for Global Health, School of Public Health, Nanjing Medical University, Nanjing, Jiangsu 211166, China; National Key Laboratory of Intelligent Tracking and Forecasting for Infectious Diseases, National Institute for Communicable Disease Control and Prevention, Chinese Center for Disease Control and Prevention, Beijing 102206, China; National Key Laboratory of Intelligent Tracking and Forecasting for Infectious Diseases, National Institute for Communicable Disease Control and Prevention, Chinese Center for Disease Control and Prevention, Beijing 102206, China; National Key Laboratory of Intelligent Tracking and Forecasting for Infectious Diseases, National Institute for Communicable Disease Control and Prevention, Chinese Center for Disease Control and Prevention, Beijing 102206, China; National Key Laboratory of Intelligent Tracking and Forecasting for Infectious Diseases, National Institute for Communicable Disease Control and Prevention, Chinese Center for Disease Control and Prevention, Beijing 102206, China; Research Units of Discovery of Unknown Bacteria and Function, Chinese Academy of Medical Sciences, Beijing 100730, China; Hebei Key Laboratory of Intractable Pathogens, Shijiazhuang Center for Disease Control and Prevention, Shijiazhuang, Hebei 050011, China; National Key Laboratory of Intelligent Tracking and Forecasting for Infectious Diseases, National Institute for Communicable Disease Control and Prevention, Chinese Center for Disease Control and Prevention, Beijing 102206, China; Department of Epidemiology, Center for Global Health, School of Public Health, Nanjing Medical University, Nanjing, Jiangsu 211166, China; Research Units of Discovery of Unknown Bacteria and Function, Chinese Academy of Medical Sciences, Beijing 100730, China

**Keywords:** Eubacterium limosum, colorectal cancer, metabolites, tumor microenvironment, gut microbiota

## Abstract

Gut microbiota play a key role in ameliorating colorectal cancer (CRC). *Eubacterium limosum* is a potential probiotic with anti-CRC functions. However, the mechanistic basis of its anti-CRC effect remains largely unknown. *In vitro*, we detected the effects of the *E. limosum* strain El1405 on cell proliferation, colony formation, cell cycle, and apoptosis of CRC cells, and found that El1405CS specifically suppressed cell proliferation by altering cell cycle distribution and inducing apoptosis. In the CT26 syngeneic mouse model, daily gavage with live El1405, inactivated El1405, culture supernatant of El1405, and El1405-derived indole derivatives, including indole-3-lactic acid (ILA), indole-3-acetic acid (IAA), L-arginine, and butyrate, inhibited tumor growth. Analysis of the 16S rRNA gene sequences revealed that El1405 altered the microbiota compositions within tumors, primarily reducing the abundance of *Enterobacter, Pseudomonas*, and *Staphylococcus*. *Staphylococcus succinus* isolated from the tumors of CT26 syngeneic mice promoted abdominal metastasis of tumors. Moreover, El1405 intervention significantly increased the levels of TNF-α, INF-γ, and CD8 in the tumor microenvironment, while decreasing the levels of CD4, IL-6, IL-10, and TGF-β*.* Metabolomic analysis indicated that El1405 induced antitumor effects through changing the serum metabolome of mice by producing indole derivatives such as ILA and IAA. Furthermore, 16S rRNA gene sequencing demonstrated that El1405 intervention changed the composition of intestinal flora, significantly increasing the abundance of *Roseburia* and *Eubacterium* while decreasing the abundance of *Staphylococcus* and *Enterococcus.* These findings suggest that *E. limosum* El1405 is a potential probiotic candidate for the prevention of CRC.

## Graphical Abstract

**Graphical Abstract:**
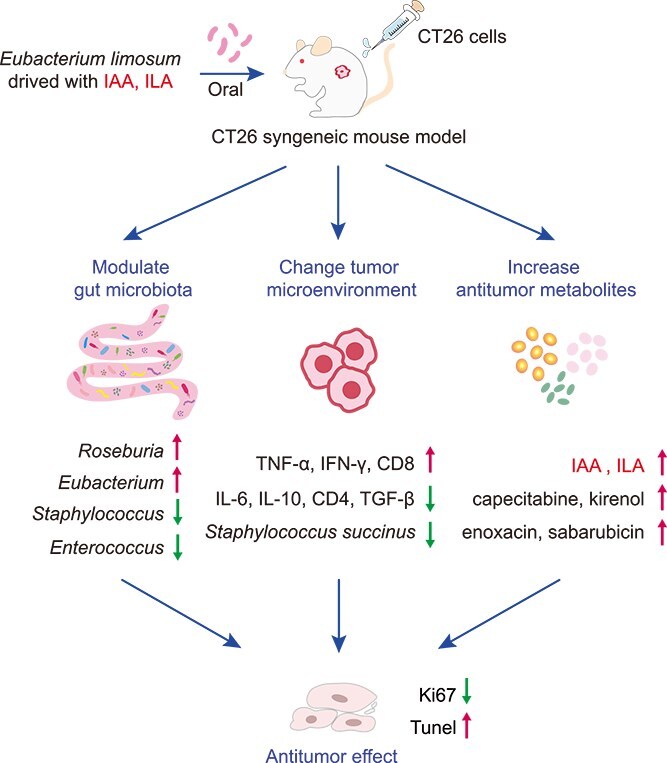

## Introduction

Colorectal cancer (CRC) is a prevalent gastrointestinal malignancy, which ranks the 3^rd^ in incidence and the 2^nd^ in mortality, and accounts for ~10% of all annually diagnosed cancers and cancer-related deaths worldwide [1]. Genetic, lifestyle, obesity, and environmental factors are the major risk factors associated with CRC carcinogenesis [2]. It has been recently recognized that gut microbiota composition is associated with CRC risks [3]. Different bacterial taxa are linked with either protecting against or promoting CRC [4]. *Fusobacterium nucleatum*, *Streptococcus bovis*, *Enterotoxigenic Bacteroides fragilis*, *Helicobacter pylori*, *Escherichia coli*, and others were associated with an increased risk of CRC [3]. Conversely, *Lactobacillus gallinarum* protected against intestinal tumorigenesis by producing protective metabolites [5]. *Clostridium butyricum* was reported to regulate anticancer drug 5-fluorouracil (5-FU) sensitization and increase the therapeutic potential of immune checkpoint inhibitors in CRC [6]. *Roseburia intestinalis* protected against CRC by producing butyrate which boosted anti-PD-1 efficacy in CRC by activating cytotoxic CD8^+^ T cells [7]. *Lactobacillus plantarum* L168 ameliorated colorectal tumorigenesis through the secretion of indole-3-lactic acid [8]. *Eubacterium callanderi* exhibited anti-CRC activity both *in vivo* and *in vitro* [9]. These studies highlight the potential of probiotics in preventing CRC and suggest their prospective value in CRC prevention and treatment.

*Eubacterium* is one of the core genera in the human gut microbiota and has been recognized as potentially beneficial microbes [10]. At the genus level, *Eubacterium* was one of the CRC-depleted taxa in the gut microbiota of CRC patients, and significantly more abundant in healthy volunteers than in CRC patients [11, 12]. Additionally, compared with nonresponders to immune checkpoint inhibitors, *Ruminococcus* spp., *Alistipes* spp., and *Eubacterium* spp. were enriched in responders [13]. These studies suggest that *Eubacterium* may play a role in inhibiting CRC.

The genus *Eubacterium* currently consists of over 40 species, with *E. limosum* being one of the major species in the human gut [10]. *E. limosum* is capable of producing short-chain fatty acids (SCFAs), including acetate and butyrate. Butyrate has been shown to significantly enhance the production of IFN-γ and TNF-α in CD8^+^ T cells, thereby increasing antitumor activity [14]. *E. limosum* has been reported to alleviate experimental colitis in mice by reducing the expression of IL-6 and TLR4 in colonocytes [15]. Recently, it has been found that *E. limosum* suppresses colorectal tumorigenesis by altering the gut microbiota profile and metabolites, with an increase in the abundance of *E. limosum* after the intervention [16]. However, the protective or ameliorative effects of *E. limosum* on the development of CRC remain to be elucidated.

In this study, we aimed to determine the mechanism of *E. limosum* involved in CRC prevention. We isolated *E. limosum* El1405 from a fecal sample of a healthy human individual and examined its antitumor effects both *in vitro* and *in vivo*. Our findings indicated that El1405 prevented CRC development by ameliorating gut dysbiosis, remodeling the tumor microenvironment (TME), and producing metabolites with antitumor properties.

## Materials and methods

### Cell culture

The HT-29, Caco-2 (human colorectal adenocarcinoma), CT26 (mouse colorectal carcinoma), NCM460 (human normal colonic epithelium), A549 (human non-small cell lung cancer), Hela (human cervical cancer), AGS and MGC-803 (human gastric cancer) cell lines used in this study were all purchased from the Cell Resource Center of Peking Union Medical College. The experiments were all completed within three to 15 generations.

### Isolation and culture of *E. limosum* strains and preparation of culture supernatant

Fresh fecal samples from healthy volunteers were collected and gradient diluted in phosphate buffered saline (PBS), and 100 μl dilutions were evenly spread on reinforced clostridial medium (RCM; Hopebio, China) agar. After anaerobic incubation at 37°C for 48–72 h, white smooth colonies were selected and subcultured on RCM plates. Purified colonies were maintained in RCM broth with 20% glycerol and stored at −80°C. Subsequently, the species identity of isolates was determined using 16S rRNA gene sequencing. Three isolates (El1405, El1408, El1739) were identified as *E. limosum*, and El1405 was selected for subsequent experiments as detailed in the results. *E. limosum* El1405 has been deposited in the China General Microbiological Culture Collection Center (CGMCC) with preservation number CGMCC NO. 31231.

El1405 was revived from −80°C and anaerobically cultured at 37°C on RCM plates for 48 hours. *E. coli* MG1655, as a negative control, was cultured under the same conditions as El1405. To prepare culture supernatant, El1405 and MG1655 (OD_600_ = 0.6, 100 μl) were inoculated into 100 ml RCM broth and incubated under anaerobic conditions for 48 h at 37°C. Then, the cells were removed by centrifugation at 3000 g for 10 min, and the supernatant was filtered through a 0.22 μM Millipore filter membrane and stored at −80°C. The supernatant of *E. limosum* El1405 was denoted as El1405CS. To remove the effect of acidic pH of El1405CS, pH was adjusted to 7.0 (RCM pH = 7.0 ± 0.1). To remove the effect of enzymatic or other activities of the proteins in the supernatant, El1405CS was heated at 100°C for 10 min or treated with Proteinase K (100 μg/ml). To explore the composition of the supernatant of El1405, El1405CS was separated with a Centrifugal concentrator, 3 kDa MWCO (Merck-Millipore). El1405CS was divided into >3-kDa and < 3-kDa parts. For heat-treated El1405 (El1405HT), El1405 was collected after 48 h of culture on RCM solid medium, and 1 × 10^8^ CFU live bacteria were suspended in PBS and heated at 70°C for 30 minutes.

### Animal experiments

Female BALB/c mice (5–6 weeks, 16–18 g) were purchased from Vital River Lab Animal Technology Co., Ltd. (Beijing, China) [laboratory animal permit no. SYXK (Jing) 2022–0029]. Mice were maintained at 23 ± 2°C, 55% ± 5% relative humidity, and a 12 h light–dark cycle under specific pathogen-free conditions. At the end of the acclimatization period, CT26 cells (5 × 10^5^ cells/mouse, cells were suspended in PBS) were subcutaneously injected into the right flank of each mouse on Day 0 to establish the CT26 syngeneic mouse model. The tumor length and width were measured by vernier caliper every 2 days, and the mice were sacrificed after 15–16 days. Tumor volume (V) was calculated by V = (length × width^2^)/2. The tumor volumes are presented as the mean ± standard error of mean (SEM).

Bacterial gavage was started at the same time as tumor inoculation on Day 0. Mice were randomized and daily gavage with (i) PBS, (ii) *E. limosum* El1405 (1 × 10^8^ CFU/0.2 ml per mouse, bacteria resuspended in PBS), (iii) *Staphylococcus succinus* (1 × 10^8^ CFU/0.2 ml per mouse, bacteria resuspended in PBS), (iv) RCM (0.2 ml per mouse), (v) El1405CS (0.2 ml per mouse), (vi) El1405HT (1 × 10^8^ CFU/0.2 ml per mouse, bacteria resuspended in PBS).

For the metabolites model, mice were daily gavaged with (i) indole-3-lactic acid (ILA) (Sigma-Aldrich, 20 mg/kg), [5] (ii) indole-3-acetic acid (IAA) (Sigma-Aldrich, 100 mg/kg), [17] (iii) L-arginine (Sigma-Aldrich, 1.5 g/kg), [18] (iv) gamma-aminobutyric acid (GABA) (Sigma-Aldrich, 100 mg/kg), [19] (v) sodium butyrate (NaBu, Sigma-Aldrich, 1 g/kg), [20] (vi) vehicle, water with 4% dimethyl sulfoxide (DMSO). The control group was treated with the vehicle of these metabolites. Metabolites were dissolved in water containing 4% DMSO. The concentration of metabolites administered in this study was selected based on previous reports.

All animal studies were approved by the Ethics Review Committee of the National Institute for Communicable Disease Control and Prevention at the Chinese Center for Disease Control and Prevention (Approval number: 2024–004).

### Statistical analysis

Differences between two groups were tested using Student’s t-test. Differences among multiple groups using one-way analysis of variance (ANOVA) test with multiple comparisons test. Tumor volume was determined by a two-way ANOVA test. All tests were performed using GraphPad Prism 9.0 software. Data were presented as mean ± SEM. *P* value <0.05 indicates statistical significance. ^*^P < .05, ^**^P < .01, ^***^P < .001, and ^****^P < .0001.

Additional methods are provided in the supplemental material.

## Results

### *E. limosum* supernatant inhibits the proliferation of CRC cells

**Figure 1:**
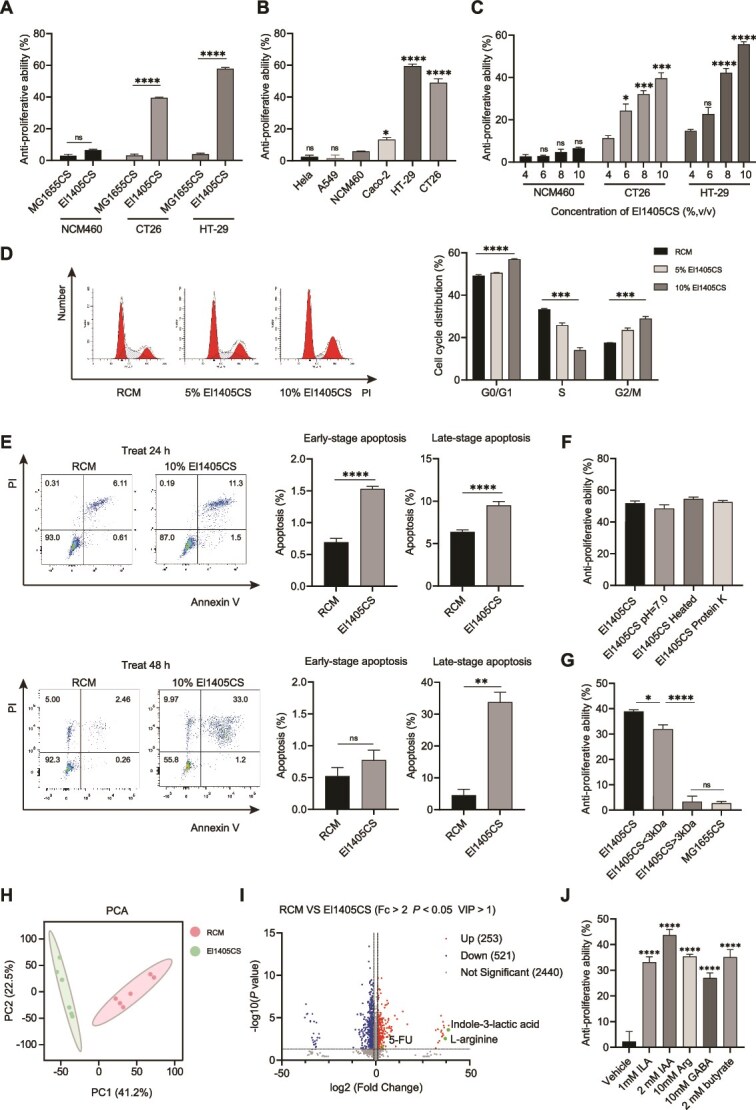
*E. limosum* supernatant inhibits the proliferation of CRC cells; (A) El1405CS (10%) significantly suppressed the cell proliferation of CT26 and HT-29. El1405CS did not exert any influence on the proliferation of the normal colonic epithelial cell line NCM460. RCM served as a blank control and was deducted from the calculation of the anti-proliferation rate; (B) El1405CS significantly suppressed the cell proliferation of CRC cell lines, whereas it did not affect other cell lines; (C) El1405CS with different concentrations reduced the CRC cell viability, except for normal epithelial cell line NCM460, and the inhibitory effect on CRC cells was dose-dependent; (D) after 24 h treatment with El1405CS (5%,10%), it induced CT26 cell cycle arrest at G0/G1 and G2/M phases; (E) the cell apoptosis of CT26 cells was significantly increased by El1405CS (10%) at 24 and 48 hours; (F) the El1405CS still exhibited inhibitory effects on the proliferation of CT26 cells after various conditions of treatment; (G) El1405CS < 3 kDa fraction reduced the CT26 cell viability; (H) based on the principal component analysis (PCA) of RCM and El1405CS, the groups of RCM and El1405CS were completely separated; (I) the volcano plot of differential metabolites content in RCM and El1405CS (Fc > 2, *P* < .05, and VIP > 1). Dots with red colors represented 253 up-regulated metabolites, and blue colors show 521 down-regulated metabolites. (n = 6 biological replicates/group); (J) antiproliferative effect of indole-3-lactic acid (ILA), indole-3-acetic acid (IAA), L-arginine (Arg), γ-aminobutyric acid (GABA), and butyrate on CT26 cell line; statistical significance was determined by unpaired two-tailed Student’s t test (A and E), one-way ANOVA with Dunnett’s multiple comparisons test (B, C, D, F, and J), and one-way ANOVA with Tukey’s multiple comparisons test (G). Data are presented as mean ± SEM. Ns, not significant; ^*^, *P* < .05; ^**^, *P* < .01; ^***^, *P* < .001, ^****^P < .0001. RCM, reinforced clostridium medium; CRC, colorectal cancer; MG1655CS, *E. coli* MG1655 culture supernatant; El1405CS, *E. limosum* El1405 culture supernatant.

Among three *E. limosum* isolates obtained from fecal samples of healthy adults, the culture supernatant of *E. limosum* El1405 (El1405CS) exhibited the strongest anti-proliferative effect on two CRC cell lines (murine CT26 and human HT-29). Therefore, El1405 was selected for further study (Fig. S1A). Compared with the non-pathogenic *E. coli* MG1655 supernatant (MG1655CS), El1405CS significantly suppressed the proliferation of CT26 and HT-29 cells, but has no effect on the normal colonic epithelial cell line NCM460 (Fig. 1A). Moreover, El1405CS mainly inhibited the proliferation of CRC cell lines such as Caco-2, CT26, and HT-29, with no effect on NCM460 and other cancer cell lines, such as Hela and A549 (Fig. 1B). The inhibitory effect of El1405CS on CRC cells was dose-dependent (Fig. 1C). Additionally, when treated with El1405CS, the number of CT26 cell colonies was significantly reduced compared with MG1655CS (Fig. S1B).

Flow cytometry showed that compared with RCM treatment, CT26 cells treated with El1405CS significantly arrested the cell cycle of G0/G1 and G2/M phases, and decreased the distribution of S phase cells at 24 hours. El1405CS treatment induced early-stage and late-stage apoptosis of CT26 cells at 24 hours, whereas it induced late-stage apoptosis of CT26 cells at 48 hours (Fig. 1D and E). Similarly, El1405CS treatment also arrested the cell cycle of G0/G1 at 24 hours and increased apoptosis in HT29 cells at 48 hours (Fig. S1C and D).

We found that there was no significant change in the ability of El1405CS to inhibit the proliferation of CRC cells following various treatments (including adjusting the pH of El1405CS from 5.1 to 7.0, heating at 100°C for 10 minutes, or treatment with protease K) (Fig. 1F and S1E). When El1405CS was divided into two fractions: > 3-kDa and < 3-kDa, the El1405CS < 3-kDa fraction had anti-proliferation ability against CT26 and HT-29, whereas the El1405CS > 3-kDa fraction did not (Fig. 1G and S1F). These findings suggest that the anti-CRC components of El1405CS may consist of metabolites with a molecular weight of <3 kDa, rather than proteins.

Potential anti-CRC metabolites in El1405CS were identified using untargeted LC–MS/MS assay. PCA indicated that the metabolite profiles of RCM and El1405CS were distinct (Fig. 1H). A total of 774 metabolites with differential concentrations were identified between RCM and El1405CS, including 253 metabolites that exhibited elevated levels and 521 metabolites that showed decreased levels in El1405CS compared to RCM (fold change >2, *P* value <0.05, VIP > 1). In particular, 5-FU, ILA, and L-arginine (Arg) were significantly elevated in El1405CS (Fig. 1I). Further targeted metabolic profiling of tryptophan, amino acids, and SCFAs (Tables S1, S2, and  S3) showed that El1405CS mainly contained ILA, IAA, Arg, GABA, and butyrate, which had anti-proliferation activity against CT26 cells (Fig. 1J).

ILA and IAA are associated with tryptophan metabolism [21]. To confirm the effect of L-tryptophan, different concentrations of tryptophan were added to the RCM medium, resulting in enhanced CT26 cell proliferation as tryptophan concentration increased (Fig. S1G). Meanwhile, the inhibitory ability of El1405CS on the proliferation of CT26 was enhanced with the increase of tryptophan concentrations in the RCM medium, whereas tryptophan did not affect the proliferation of NCM460 (Fig. S1H and I). Thus, a portion of the anti-CRC effect exhibited by El1405 was related to tryptophan metabolism. Moreover, ILA and IAA are downstream metabolites of L-tryptophan, which usually exert their effects through their receptor, aryl hydrocarbon receptor (AhR) [21]. Pre-treatment of CT26 cells with the AhR-specific antagonist (CH223191) for 12 hours partially abolished the anti-cell-proliferative effect of El1405CS (Fig. S1J), indicating that the anti-CRC effect of El1405 was associated with the downstream metabolites of L-tryptophan. Collectively, these data suggested that El1405CS exhibited anti-CRC cell proliferative ability by producing metabolites with anti-CRC properties.

### Genomic evidence of *E. limosum-*producing metabolites

**Figure 2:**
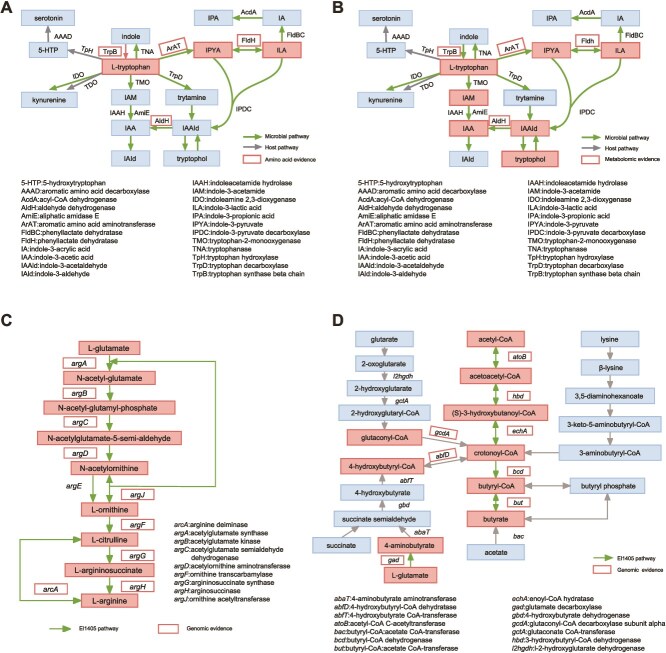
Genomic and metabolome analyses of *E. limosum*. The *E. limosum* genome is enriched in genes involved in the catabolism of tryptophan, synthesis of arginine, synthesis of γ-aminobutyric acid, and synthesis of butyrate; (A) pathway schematic of bacterial and abbreviated mammalian tryptophan metabolism. The El1405 genome is enriched in genes involved in the catabolism of tryptophan. Enzymes with amino acid evidence in El1405 are boxed in red, including ArAT and FldH; (B) metabolome analysis of El1405. El1405 produces a wide array of tryptophan-derived metabolites known to modulate the immune system. Compared to RCM, the tryptophan-derived metabolites produced by El1405 are boxed in red; (C) the El1405 genome contains all synthetic genes from L-glutamate to L-arginine. Relevant genes are boxed in red; (D) the El1405 genome contains multiple genes related to the butyric acid production pathway, mainly through the acetyl-CoA pathway to produce butyrate. El1405 primarily utilizes butyryl-CoA: Acetate CoA transferase to transfer coenzyme A from butyryl-coenzyme to acetate to form butyrate. Relevant genes are boxed in red.

To determine whether El1405 encodes the genes involved in the catabolism of above detected metabolite, genomic analysis of El1405 was performed. The El1405 genome contained genes encoding phenyllactate dehydrogenase (FldH) (41.8% identity, 97.3% coverage), tryptophan synthase beta chain (TrpB) (64.9% identity, 96.7% coverage), aldehyde dehydrogenase (AldH) (99.4% identity, 99.8% coverage), and aromatic amino acid aminotransferase (ArAT) (100% identity, 99.8% coverage), which are required for the production of indole derivatives (Fig. 2A). These findings indicated that El1405 contains genes that are associated with tryptophan metabolism. Metabolomic analysis of El1405 revealed that El1405 produced a range of tryptophan-derived metabolites, including ILA, IAA, indole-3-acetamide (IAM), and tryptophol (Fig. 2B). Collectively, these data suggest that El1405 encodes genes essential for metabolizing host dietary tryptophan to produce indole derivatives.

The genome of El1405 was found to contain all the genes required for the synthesis of L-arginine from L-glutamate (Fig. 2C), the gene encoding glutamate decarboxylase which acts on L-glutamate to form GABA and multiple genes related to the butyric acid production pathway, mainly through the acetyl-CoA pathway to produce butyrate (Fig. 2D). These results suggested that El1405 directly contributes to L-arginine, GABA, and butyrate production.

### *E. limosum* inhibits tumor growth in the CT26 syngeneic mouse model

**Figure 3:**
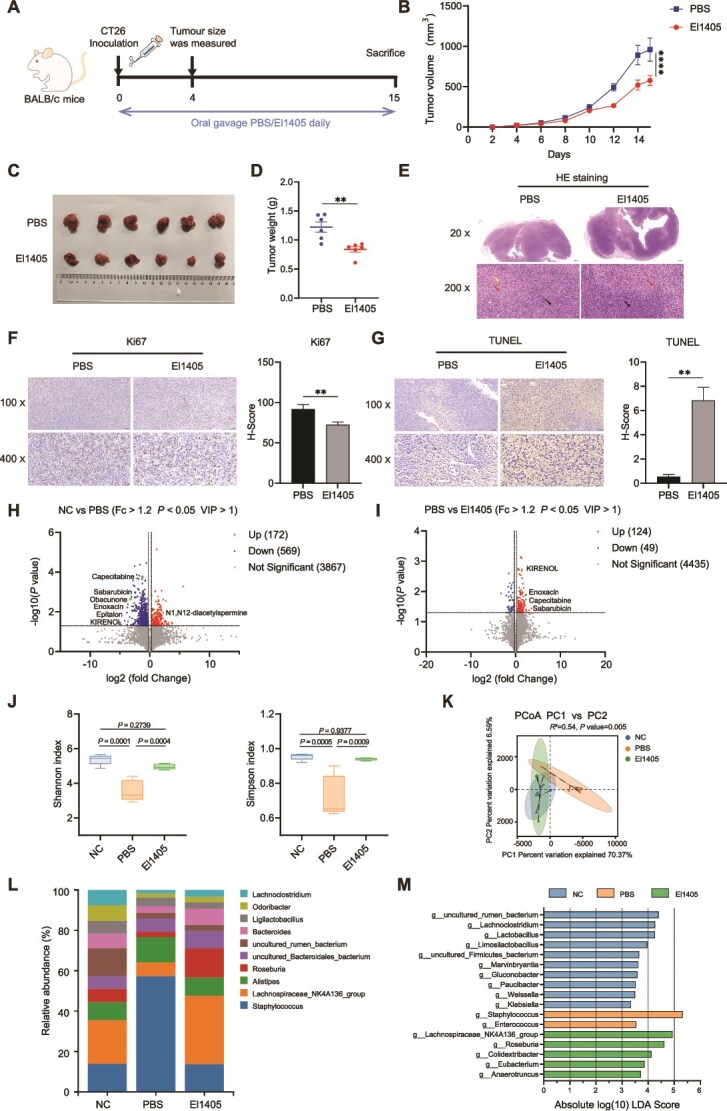
*E. limosum* inhibits tumor growth in the CT26 syngeneic mouse model; (A) schematic diagram of the CT26 syngeneic mouse model and El1405 treatment schedule; (B) Oral gavage El1405 daily, significantly inhibited tumor growth, as evidenced by tumor volume; (C) representative tumor picture; (D) tumor weight in the CT26 syngeneic mouse model (n = 6); (E) representative images of tumor stained with HE. Scale bar: 20×, 500 μm; 200×, 50 μm. The black arrows indicate the nuclear division, the red arrows indicate the necrosis area, and the yellow arrow indicates the inflammatory infiltration; (F) IHC staining revealed that El1405 supplementation reduced Ki67 expression in tumor tissues (n = 3). Scale bar: 100×, 100 μm; 400×, 20 μm; (G) IHC staining revealed that El1405 supplementation increased TUNEL expression in tumor tissues (n = 3). Scale bar: 100×, 100 μm; 400×, 20 μm; (H) through the untargeted metabolomics analysis, the volcano plot of differential metabolites in gut contents of NC and PBS group (fc > 1.2, *P* < .05, and VIP > 1). Dots with red colors represent the 172 up-regulated metabolites, and blue colors show the 569 down-regulated metabolites (n = 5); (I) through untargeted metabolomics, the volcano plot of differential metabolites in gut contents of PBS and El1405 group (fc > 1.2, *P* < .05, and VIP > 1). Dots with red colors represent the 124 up-regulated metabolites, and blue colors show the 49 down-regulated metabolites (n = 5); (J) boxplots of alpha diversity index among three groups. This was shown by the Shannon and Simpson indices; (K) PCoA plots based on Euclidean distance; (L) Barplot analysis of microbiota composition profiling at the genus level (top 10); (M) overrepresented bacterial taxa among groups determined by LDA score with a threshold of 3; the statistical significance of the tumor volume was determined by two-way ANOVA test. Statistical significance was determined by unpaired two-tailed student t-test (D, F, and G) and one-way ANOVA with Tukey’s multiple comparisons test (J). Data are presented as mean ± SEM. ns, not significant; ^*^, *P* < .05; ^**^, *P* < .01; ^***^, *P* < .001, ^****^P < .0001. NC, negative control; PBS, phosphate-buffered saline; HE, hematoxylin–eosin staining; IHC, immunohistochemistry.

To investigate the antitumor effect of El1405 *in vivo,* a syngeneic mouse model was established (Fig. 3A). Two groups of mice were injected with CT26 cells, with one group treated with El1405 (the El1405 group) and the other with PBS (the PBS group). Additionally, a third group of mice that did not receive any treatment and were not injected with CT26 cells served as a negative control (the NC group). Supplementation with El1405 significantly inhibited tumor growth and reduced tumor volume, size, and weight compared to the PBS group in the CT26 syngeneic mouse model (Fig. 3B-D). Hematoxylin–eosin (HE) staining revealed that tumor cells in the PBS group had a higher rate of nuclear division and multiple hemorrhages compared to the El1405 group (Fig. 3E). However, both groups displayed small areas of necrosis. Subsequent immunohistochemistry analysis indicated that treatment with El1405 reduced Ki67 expression while increasing TUNEL expression in tumors (Fig. 3F and G). Untargeted LC–MS/MS assay was used to identify metabolites in cecal contents, which showed that the concentrations of capecitabine, sabarubicin, obacunone, enoxacin, epitalon, and kirenol in the PBS group were lower than those in the NC group (Fold change >1.2, *P* value <0.05, VIP > 1). In contrast, capecitabine, sabarubicin, enoxacin, and kirenol, which were associated with antitumor properties [22–31], were higher in the El1405 group (Fig. 3H and I). In addition, levels of N1, N12-diacetylspermine, which are involved in promoting cancer cell proliferation [32, 33], were higher in the PBS group compared to the NC group.

Analysis of 16S rRNA gene sequences from mouse cecal contents revealed a significant increase in microbial abundance in the El1405 group compared to the PBS group, whereas there was no significant difference between the El1405 and the NC groups in terms of alpha diversity (Fig. 3J). Beta-diversity analysis showed that the El1405 and NC groups had similar microbiota compositions, whereas the PBS group clustered separately from these two groups (Fig. 3K). *Staphylococcus* had the highest relative abundance (57.4%) in the PBS group, whereas the *Lachnospiraceae_*NK4A136_group was the predominant genus in both the NC and El1405 groups (21.6% and 33.9%, respectively) (Fig. 3L). Additionally, *Staphylococcus* ranked third in relative abundance within the NC and El1405 groups (14.0% and 13.7%, respectively).

LEfSe also showed that pathogenic genera, such as *Staphylococcus* and *Enterococcus*, were significantly more abundant in PBS-treated mice than in the other groups [34–36] (Fig. 3M). In contrast, daily gavage with El1405 increased the abundance of *Lachnospiraceae* NK4A136, *Roseburia*, and *Eubacterium*. Some species in these two genera have been identified as butyrate producers [37]. Moreover, the abundance of *E. limosum* was significantly higher in the cecal contents of the El1405 group than in the PBS group as expected (Fig. S2A).

### Supplementation with *E. limosum* remodels the tumor microenvironment

**Figure 4:**
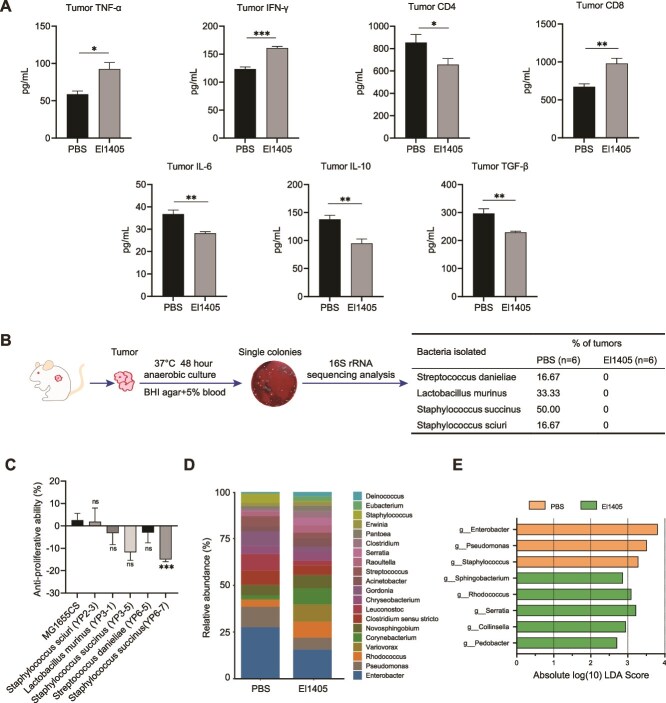
Daily supplementation with *E. limosum* altered the tumor microenvironment in the CT26 syngeneic mouse model; (A) ELISA for TNF-α, IFN-γ, CD4, CD8, IL-6, IL-10, and TGF-β in the tumor tissue; (B) schematic diagram of intratumor bacteria isolation, showing representative images of the intratumor microbe and listing live bacterial species isolated from mouse tumors; (C) MTT assays were performed to detect the anti-proliferative ability of culture supernatant of intratumor bacteria; (D) Barplot analysis of intratumor microbiota composition profiling at the genus level (top 20); (E) overrepresented bacterial taxa among groups determined by LDA score with a threshold of 2.5; statistical significance was determined by unpaired two-tailed student t-test (A) and one-way ANOVA with Dunnett’s multiple comparisons test (C). Data are presented as mean ± SEM. ns, not significant; ^*^, *P* < .05; ^**^, *P* < .01; ^***^, *P* < .001, ^****^P < .0001. TNF-α, tumor necrosis factor-α; IFN-γ, interferon-γ; IL-6, Interleukin-6; IL-10, Interleukin-10; TGF-β, transforming growth factor-β; MTT, 3-(4,5-dimethyl-2-thiazolyl)-2,5-diphenyl-2-H-tetrazolium bromide.

TME contains various types of immune cells that play a significant role in tumor development [38]. To evaluate El1405-mediated immunomodulation within the TME, soluble T cell activity markers (CD4/CD8) and cytokines were quantified by ELISA. Compared with the PBS group, the El1405 group significantly increased the levels of antitumor effectors, such as TNF-α, INF-γ, and cytotoxic T cell-associated CD8, and significantly decreased the levels of cancer-promoting and immunosuppressive cytokines (IL-6, IL-10, and TGF-β) and regulatory T cell-linked CD4 (Fig. 4A).

Live bacteria have been found in various tumors [39], influencing tumor development and treatment through diverse mechanisms [40]. We isolated live bacteria from tumors under anaerobic conditions and identified four species in the tumors of the PBS group: *S. succinus*, *Streptococcus danieliae*, *Lactobacillus murinus*, and *Staphylococcus sciuri*, whereas no live bacteria were isolated in the El1405 group (Fig. 4B). *S. succinus* constituted 50% of the total isolates. These four species were cultured under anaerobic conditions in a brain heart infusion medium, and their supernatants were collected and tested with CT26 cells respectively. The results showed that only the supernatant of *S. succinus* promoted the proliferation of CRC CT26 cells (Fig. 4C).

Sequencing of the 16S rRNA genes was performed on the tumors to investigate changes in intratumoral microbes. No significant difference was found in microbial abundance between the El1405 and PBS groups (Fig. S2B). However, *Enterobacter* exhibited the highest relative abundance in both the PBS and El1405 groups (27.6% and 15.8% respectively) (Fig. 4D). In the PBS group, *Pseudomonas* was the second most abundant genus (11.0%), whereas *Variovorax* ranked as the second predominant genus (9.3%) in the El1405 group. Analysis of intratumoral microbes using LEfSe revealed that the abundance of *Enterobacter, Pseudomonas, and Staphylococcus* was significantly higher in PBS-treated mice than in the El1405 group. Conversely, in the El1405 group the abundance of *Collinsella* was increased (Fig. 4E), some species of which have been reported as probiotics [41, 42].

### Inactivated *E. Limosum* inhibits tumor growth in the CT26 syngeneic mouse model

**Figure 5:**
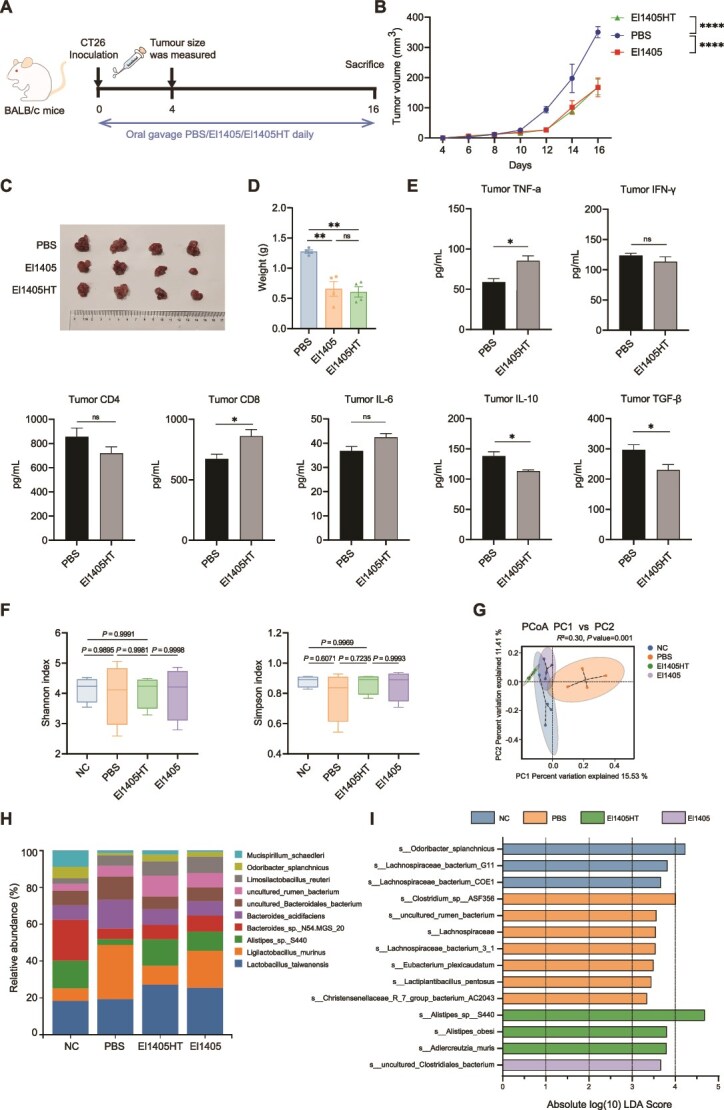
Inactivated *E. limosum* inhibits tumor growth in the CT26 syngeneic mouse model; (A) schematic diagram of the CT26 syngeneic mouse model and El1405HT gavage schedule; (B) Oral gavage of El1405HT daily, significantly inhibited tumor growth, as evidenced by tumor volume; (C) representative tumor picture; (D) tumor weight in the CT26 syngeneic mouse model (n = 4); (E) ELISA for TNF-α, IFN-γ, CD4, CD8, IL-6, IL-10, and TGF-β in the tumor tissue; (F) boxplots of alpha diversity index among the four groups. This was shown by the Shannon and Simpson indices; (G) PCoA plots based on binary Jaccard distance; (H) Barplot analysis of microbiota composition profiling at the species level (top 10); (I) overrepresented bacterial taxa among groups determined by LDA score with a threshold of 3; the statistical significance of the tumor volume was determined by two-way ANOVA test. Statistical significance was determined by unpaired two-tailed student t-test (E) and one-way ANOVA with Tukey’s multiple comparisons test (D and F). Data are presented as mean ± SEM. ns, not significant; ^*^, *P* < .05; ^**^, *P* < .01; ^***^, *P* < .001, ^****^P < .0001. El1405HT, heat-treated El1405 (heated at 70°C for 30 minutes).

We tested the antitumor effect of inactivated El1405 (El1405HT) *in vivo* (Fig. 5A) and showed that supplementation with El1405HT significantly inhibited tumor growth and reduced tumor volume, size, and weight compared to the PBS group in the CT26 syngeneic mouse model (Fig. 5B-D). There was no significant difference in tumor weight between the El1405HT group and the El1405 group. Compared with the PBS group, the El1405HT group significantly increased the levels of TNF-α and CD8 in TME, and decreased the levels of IL-10 and TGF-β (Fig. 5E).

Analysis of the 16S rRNA gene sequences from mouse cecal contents revealed that there was no significant difference in microbial abundance among the NC, PBS, El1405HT*,* and El1405 groups (Fig. 5F). Beta-diversity analysis revealed that the NC, El1405HT, and El1405 groups had similar microbial compositions, whereas the PBS group clustered separately from these three groups (Fig. 5G). *L. murinus* showed the highest relative abundance (29.3%) in the PBS group, whereas *Lactobacillus taiwanensis* was the predominant species in the El1405HT and El1405 groups (27.2% and 25.6%, respectively) (Fig. 5H). Analysis of intestinal microbiota composition using LEfSe showed that the abundance of pathogenic species, such as *Clostridium* sp. ASF356 was significantly higher in PBS-treated mice than in the other groups [43] (Fig. 5I). Conversely, the abundance of *Alistipes obesi* was increased in the El1405 group compared to the other groups.

### *E. limosum* supernatants inhibited tumor growth in the CT26 syngeneic mouse model

**Figure 6:**
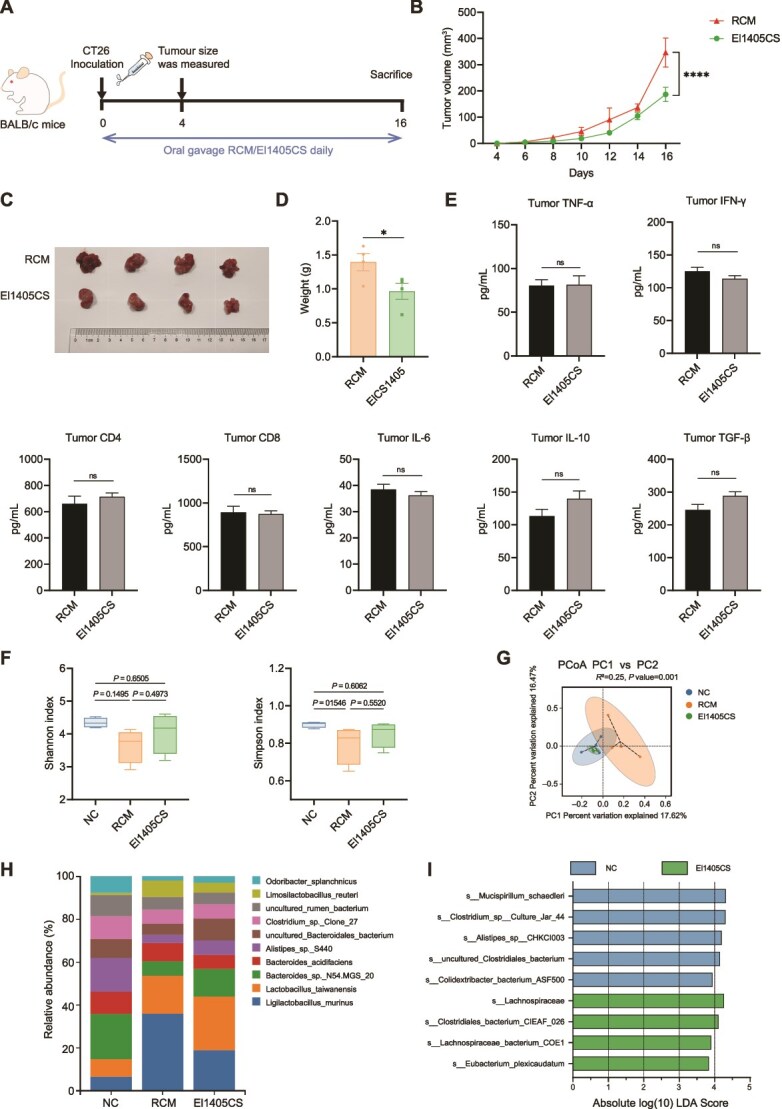
*E. limosum* supernatants inhibited tumor growth in the CT26 syngeneic mouse model; (A) schematic diagram of the CT26 syngeneic mouse model and El1405CS gavage schedule; (B) Oral gavage of El1405CS daily, significantly inhibited tumor growth, as evidenced by tumor volume; (C) representative tumor picture; (D) tumor weight in the CT26 syngeneic mouse model (n = 4); (E) ELISA for TNF-α, IFN-γ, CD4, CD8, IL-6, IL-10, and TGF-β in the tumor tissue; (F) boxplots of alpha diversity index among three groups. This was shown by the Shannon and Simpson index; (G) PCoA plots based on binary Jaccard distance; (H) Barplot analysis of microbiota composition profiling at the species level (top 10); (I) overrepresented bacterial taxa among groups determined by LDA score with a threshold of 3. The statistical significance of the tumor volume was determined by two-way ANOVA test. Statistical significance was determined by unpaired two-tailed Student t-test (E) and one-way ANOVA with Dunnett’s or Tukey’s multiple comparisons test (D and F). Data are presented as mean ± SEM. ns, not significant; ^*^, *P* < .05; ^**^, *P* < .01; ^***^, *P* < .001, ^****^P < .0001. RCM, Reinforced Clostridium Medium; El1405CS, *E. limosum* El1405 culture supernatant.

To investigate the antitumor effect of El1405CS *in vivo*, two groups of mice were injected with CT26 cells with one group treated with El1405CS (the El1405CS group) while the other with RCM (the RCM group), the third group of mice that did not receive any treatment and was not injected with CT26 cells, serving as the negative control (the NC group) (Fig. 6A). Supplementation with El1405CS significantly inhibited tumor growth and reduced tumor volume, size, and weight compared to the RCM group in the CT26 syngeneic mouse model (Fig. 6B-D). However, the levels of cytokines in TME showed no significant differences between the RCM and El1405CS groups (Fig. 6E).

Analysis of the 16S rRNA gene sequences from mouse cecal contents revealed that there was no significant difference in microbial abundance among the NC, RCM, and El1405CS groups (Fig. 6F). Beta-diversity analysis revealed that the NC and El1405CS groups had similar microbiota compositions, whereas the RCM group clustered separately from the other groups (Fig. 6G). *L. murinus* showed the highest relative abundance (36.0%) in the RCM group, whereas *L. taiwanensis* was the predominant species (25.0%) in the El1405CS group (Fig. 6H). In the NC group, *Bacteroides* sp. N54.MGS 20 was the dominant species (21.2%). LEfSe analysis showed that the El1405CS group had an increase in the abundance of *Eubacterium plexicaudatum* and *Lachnospiraceae* (Fig. 6I). The microbiota composition of live El1405, El1405HT, and El1405CS groups was comparable (Fig. S2C and D).

### Antitumor effect of metabolites in the CT26 syngeneic mouse model

**Figure 7:**
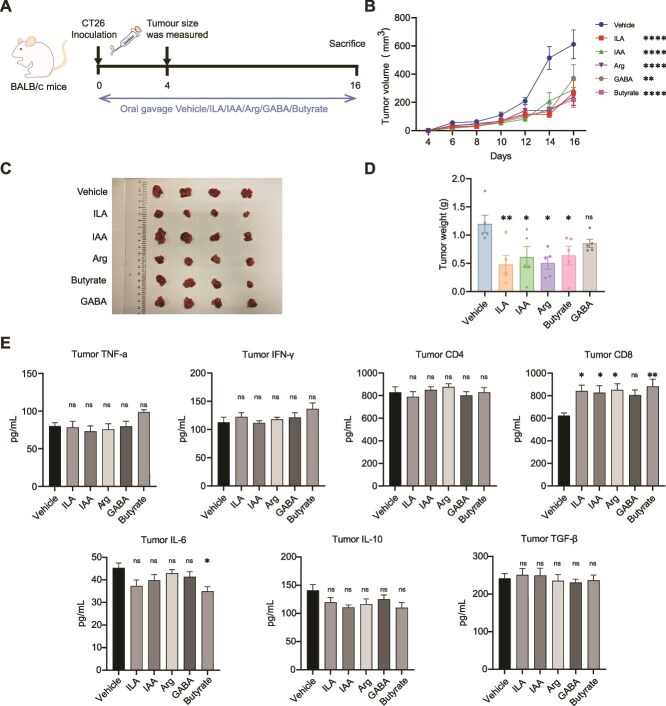
Antitumor effect of metabolites in the CT26 syngeneic mouse model; (A) schematic diagram of the CT26 syngeneic mouse model and metabolites gavage schedule; (B) Oral gavage of metabolites daily, ILA, IAA, Arg, and butyrate significantly inhibited tumor growth, as evidenced by tumor volume; (C) representative tumor picture; (D) tumor weight in the CT26 syngeneic mouse model (n = 5); (E) ELISA for TNF-α, IFN-γ, CD4, CD8, IL-6, IL-10, and TGF-β in the tumor tissue; the statistical significance of the tumor volume was determined by two-way ANOVA test. Statistical significance was determined by one-way ANOVA with Dunnett’s multiple comparisons test (D and E). Data are presented as mean ± SEM. ns, not significant; ^*^, *P* < .05; ^**^, *P* < .01; ^***^, *P* < .001, ^****^P < .0001.

The antitumor effect of the metabolites of El1405 was investigated by using the CT26 syngeneic mouse model (Fig. 7A). The control group was treated with the vehicle of these metabolites. The results showed that supplementation with ILA, IAA, Arg, or butyrate significantly inhibited tumor growth and reduced tumor volume, size, and weight compared to the vehicle group in the CT26 syngeneic mouse model (Fig. 7B-D). Further, compared with the vehicle group, the ILA, IAA, Arg, and butyrate groups significantly increased the CD8 levels in tumors (Fig. 7E). Additionally, only the butyrate group showed a significant decrease in IL-6 levels.

### *E. limosum* altered the metabolism of serum in the CT26 syngeneic mouse model

**Figure 8:**
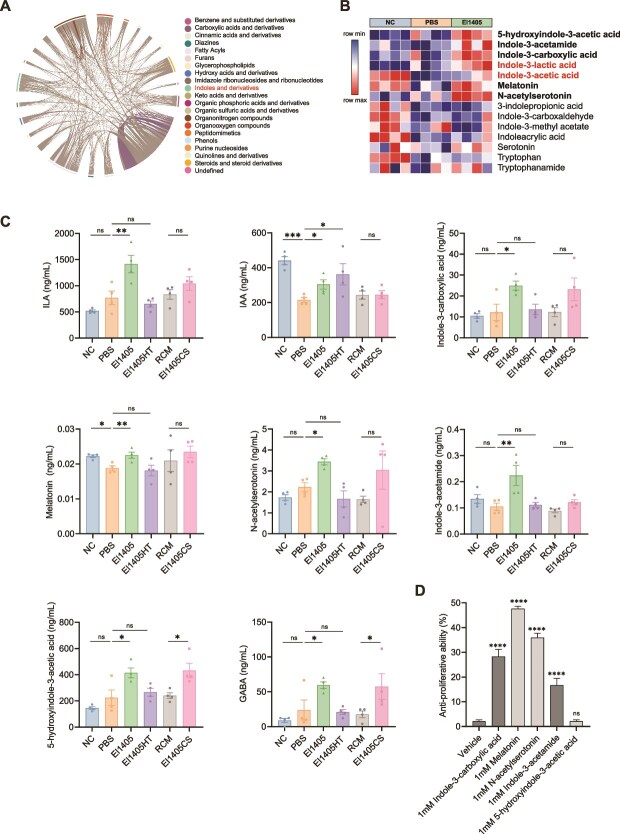
*E. limosum* altered the metabolism of serum in the CT26 syngeneic mouse model; (A) the chord diagram showed the correlation between various metabolites, |r| > 0.8, *P* < .05 (n = 4). Pearson’s correlation analysis was performed to determine the correlation among all variables; (B) Heatmap of indole derivatives from serum samples. (n = 4); (C) metabolites enriched in serum samples of El1405-treated mice (n = 4); (D) Antiproliferative effects of various metabolites on CT26 cell line; statistical significance was determined by one-way ANOVA test with Dunnett’s multiple comparisons test (C and D). Data are presented as mean ± SEM. ns, not significant; ^*^, *P* < .05; ^**^, *P* < .01; ^***^, *P* < .001, ^****^P < .0001.

The serum metabolites of mice were analyzed by targeted metabolomics. Orthogonal partial least squares-discriminant analysis (OPLS-DA) revealed distinct serum metabolite profiles between the PBS and El1405 groups (Fig. S2E). A total of 342 metabolites were identified, which were quantified and classified into 40 categories based on their chemical taxonomy. Among these, indole and its derivatives accounted for 4.09% of the total metabolites (Fig. S2F). Correlation analysis demonstrated the relationships between significantly differential metabolites using a chord diagram, highlighting metabolite pairs with a correlation coefficient (|r| > 0.8 and *P* < 0.05) (Fig. 8A). This method identified 343 metabolites and revealed that 76 metabolites showed different concentrations between the PBS and El1405 groups, including 74 metabolites that exhibited elevated levels and 2 metabolites that showed decreased levels in the El1405 group compared to the PBS group (Fold change >1, *P* value <0.05) (Table S4). The heatmap revealed a significant enrichment of various indole metabolites, indicating a differential abundance of indole derivatives between PBS-treated and El1405-treated mice, particularly IAA and ILA, which were also significantly elevated in the culture supernatant of El1405 (Fig. 8B).

The serum levels of ILA, IAA, indole-3-carboxylic acid (ICA), melatonin, N-acetylserotonin, IAM, 5-hydroxyindole-3-acetic acid (5-HIAA), and GABA in mice treated with El1405 live bacteria were significantly higher than those in PBS-treated mice (Fig. 8C). And the El1405HT group increased IAA levels compared to the PBS group. The El1405CS group significantly increased 5-HIAA, GABA, docosahexaenoic acid (DHA), and anandamide levels, compared to the RCM group (Fig. S2G and H). Moreover, when treated with these metabolites, it was found that ICA, IAM, melatonin, and N-acetylserotonin effectively inhibited the proliferation of CT26 cells (Fig. 8D).

### *S. succinus* promotes abdominal metastasis of the tumor in the CT26 syngeneic mouse model

The effect of *S. succinus* on tumors was further examined using the CT26 syngeneic mouse model (Fig. S3A). The results showed that the abdominal metastasis ratio of tumors in the *S. succinus* group (75%) was significantly higher than that in the PBS group (25%), but the tumor volume, size, and weight were comparable between the *S. succinus* group and the PBS group (Fig. S3 B-E). Vascular endothelial growth factor (VEGF) and its receptors are associated with carcinogenesis, invasion, and tumor angiogenesis [44]. The level of VEGF in the tumors was significantly higher in the *S. succinus* group compared to that in the PBS group (Fig. S3F).

Analysis of the 16S rRNA gene sequences from the mouse cecal contents revealed that there was no significant difference in microbial abundance among the NC, PBS, and *S. succinus* groups (Fig. S3G). Beta-diversity analysis revealed that the *S. succinus* and PBS groups had similar microbiota compositions, whereas the NC group clustered separately from these two groups (Fig. S3H). *L. taiwanensis* showed the highest relative abundance (25.1%) in the PBS group, whereas *L. murinus* was the predominant species (31.7%) in the *S. succinus* group (Fig. S3I). LEfSe showed that the abundances of pathogenic genera such as *S. succinus, Clostridioides difficile, *and* Mammaliicoccus sciuri* were significantly higher in *S. succinus*-treated mice than in the PBS group [45–47] (Fig. S3J).

## Discussion

Previous studies have found that *Eubacterium* is significantly lower in CRC patients than in healthy volunteers, whereas *Escherichia*/*Shigella*, *Enterococcus*, and *Streptococcus* are significantly higher in CRC patients [11]. *E. limosum* is one of the major *Eubacterium* species in the human gut, and previous studies have revealed the anti-inflammatory effect of *E. limosum* [15]. In this study, we demonstrated that El1405CS specifically suppressed cell proliferation by altering cell cycle distribution and inducing apoptosis in CT26 cells. In the CT26 syngeneic mouse model, daily gavage with El1405 (including live El1405, inactivated El1405, and culture supernatant of El1405) inhibited tumor growth; furthermore, treatment with live El1405 increased serum levels of indole derivatives, which have been shown to suppress tumor growth [5]. Our study confirms that *E. limosum* is a potential probiotic with CRC-suppressive effects.

Recently, it has been reported that microbial derived metabolites play key roles in regulating anti-tumor response [48]. For instance, ILA derived from *L. plantarum* ameliorated colorectal tumorigenesis [8], and 3-IAA enhanced the chemotherapy efficacy of pancreatic cancer [49]. Similarly, arginine decreased the immunosuppression of regulatory T cells (Treg cells), thereby potentiating CD8^+^ T cell-mediated antitumor activity and reducing tumor burden [50]. Butyrate augmented anti-PD-1 therapy by enhancing T cell receptor signaling in cytotoxic CD8^+^ T cells [14]. GABA has been reported to inhibit proliferation and increase anticancer drug oxaliplatin sensitivity in human CRC cells [51]. Similarly, our study demonstrates that metabolites produced by El1405, including ILA, IAA, Arg, GABA, and butyrate, inhibited the proliferation of CT26 cells and the growth of tumors. Although the exact mechanism remains unclear, we observed elevated intratumoral CD8^+^ T cell levels in mice treated with ILA, IAA, Arg, or butyrate, suggesting a potential link between these metabolites and enhanced CD8^+^ T cell infiltration in the TME. However, GABA did not exhibit this immunomodulatory effect, implying distinct pathways for its antitumor activity.

ArAT is a key enzyme in tryptophan metabolism [52]. The ArAT of El1405 exhibits 100% identity to the ArAT amino acid sequence of *E. limosum* in the UniProt database. However, there are currently no studies on the role of ArAT in tryptophan metabolism in *E. limosum*. ArAT, which converts tryptophan into indole-3-pyruvate, is enriched in the genome of *Lactobacillus reuteri* [52]. The El1405 ArAT had an amino acid sequence identity of 34.2% and a coverage of 98% compared to ArAT of *L. reuteri*. Generally, proteins with ~40% or more sequence identity are considered to perform similar functions, whereas broad functional class is conserved to ~25% [53]. Thus, it is likely that El1405 is actively involved in tryptophan metabolism.

Indole derivatives, including ILA and IAA, are downstream metabolites of L-tryptophan, which usually exert their effects through their receptor AhR [21]. Pretreatment of CT26 cells with the AhR-specific antagonist (CH223191) partially abolished the anti-cell-proliferative effect of El1405CS, indicating that the anti-CRC effect of El1405CS was associated with indole derivatives. ILA and IAA were significantly enriched in both the culture supernatant of El1405 and the serum of El1405-treated mice. This implies that ILA and IAA may be the key metabolites responsible for the anti-CRC tumor effect of El1405. In addition, in the CT26 syngeneic mouse model, the addition of El1405 also increased serum levels of ICA, IAM, melatonin, N-acetylserotonin, and 5-HIAA. Moreover, ICA, IAM, melatonin, and N-acetylserotonin exhibited anti-proliferative effects on CT26 cells. A previous study showed that in the AOM/DSS-induced CRC mouse model, supplementation with *L. gallinarum* increases ILA levels in feces and ICA levels in serum and tumor, thereby modulating antitumor immunity [5, 54]. Melatonin has been utilized as an adjunct in CRC treatment, and N-acetylserotonin is an intermediate in converting serotonin to melatonin [55, 56].

Compared to the control group, live El1405 induced a significant increase in multiple indole derivatives (such as IAA and ILA). In contrast, El1405HT only increased IAA, whereas El1405CS exclusively elevated the level of 5-HIAA. This indicates that the complete bacterial viability of El1405 is crucial for coordinating microbial tryptophan metabolism. In addition, the levels of 5-HIAA, GABA, DHA, and anandamide were also increased in the serum of mice treated with El1405CS. 5-HIAA did not suppress the proliferation of CT26 cells, and though GABA showed an anti-proliferative effect *in vitro*, it had no obvious inhibitory effect on tumor growth *in vivo*. Studies have demonstrated that DHA promotes cell cycle arrest and decreases proliferation in CRC cells [57, 58], and DHA reverses epithelial-mesenchymal transition (EMT) through the TGF-β1/Smad signaling pathway, inhibiting the invasion and migration of CRC [59]. Anandamide induces cell death in colorectal carcinoma cells by cyclooxygenase 2 [60]. Based on these studies, we speculate that the El1405CS exerts an anti-tumor effect by its specific metabolites to inhibit the growth of CRC tumors and promote cell apoptosis.

Our study found that 5-FU was present in the supernatant of El1405. Although there had been no reports that bacteria can synthesize 5-FU through natural metabolic pathways, our findings raise this possibility. However, further studies are required. Untargeted metabolomics analysis of the intestinal contents in the CT26 syngeneic mice indicated that El1405 intervention increased the levels of capecitabine, sabarubicin, enoxacin, and kirenol related to anti-tumor in the gut [22–31]. Capecitabine can enhance the therapeutic efficacy and survival rates of patients with CRC through monotherapy or combination therapy [23, 24]. The combination of sabarubicin and cisplatin exhibited strong antitumor activity in human lung tumor xenograft [22]. As a cancer-specific growth inhibitor, enoxacin can enhance microRNA processing [29, 30]. Kirenol significantly reduced the tumor volume and incidence of gastric cancer in rat models [31]. The biosynthetic sources of these anti-cancer metabolites produced in the intestines of mice after El1405 intervention remain unknown. Sabarubicin is an analogue of doxorubicin which is produced by *Actinomycetes* such as *Streptomyces peucetius* [61, 62] and is likely a microbial metabolite. However, whether capecitabine, enoxacin, and kirenol are host-derived, microbial-produced, or from other sources requires further study.

The occurrence of CRC is closely related to the intestinal microbiota. Studies have demonstrated that several pathogenic species were enriched in CRC patients, including *F. nucleatum*, *Peptostreptococcus anaerobius*, and *Peptostreptococcus stomatis* [11, 12, 63]. Transplantation of feces from CRC patients promoted tumorigenesis in germ-free or gnotobiotic mouse models and *Apc*^Min/+^ mice [64, 65]. Numerous studies have demonstrated that microbial dysbiosis contributes to cancer susceptibility through various pathways [66, 67], while the supplementation of specific microbiota helps activate an antitumor immune response [68]. Probiotics can mitigate inflammation, restore gut dysbiosis by inhibiting pathobionts while promoting beneficial commensals, and reactivate antitumor immunity, ultimately aiding in the clearance of premalignant cells [63]. *Companilactobacillus crustorum* MN047 can alleviate colitis-associated tumorigenesis by modulating the intestinal microenvironment [69]. *C. butyricum* inhibits intestinal tumor development by modulating Wnt signaling and gut microbiota [70]. In this study, El1405 intervention changed the composition of intestinal flora, significantly increased the abundance of *Lachnospiraceae* NK4A136, *Roseburia,* and *Eubacterium* while decreased the abundance of *Staphylococcus* and *Enterococcus. Lachnospiraceae* NK4A136 is important in helping maintain intestinal homeostasis [71]. Certain species of *Roseburia* and *Eubacterium* are producers of butyrate, an effective anti-cancer metabolite that promotes histone acetylation and the expression of tumor suppressor proteins [37, 72]. Previous studies have indicated that gut microbial metabolite butyrate could indirectly inhibit tumors by modulating CD8^+^ T and Treg cells [20]. In contrast, *Enterococcus faecalis* and *Staphylococcus aureus* were positively correlated with the development of tumors [73–75]. Although causality has not been directly verified, this association suggests that metabolites of the microbiota may be a potential mechanism that mediates antitumor effects.

TME cells and their secreted molecules play critical roles in the pathogenesis of cancer, making them attractive therapeutic targets [76]. CD8^+^ T cells elicit antitumor immunity through the release of TNF-α and IFN-γ, enhancing the immune response within TME [77–80]. CD4^+^ T cells can differentiate into a variety of subsets, including T_H_1, T_H_2, T_H_17, and Treg cells. In tumor immunity, CD4^+^ T cells exhibit both antitumor and protumor effects [81]. Treg cells include typical suppressive factors such as TGF-β and IL-10, which enhance the immunosuppressive capacity [82, 83]. IL-6 maintains a pro-tumor environment by supporting angiogenesis and tumor evasion of immune surveillance [84]. A recent study demonstrated that the combined treatment of IL-2 and *Akkermansia muciniphila* induces alterations in immune factors within the TME, mainly increasing the proportions of IFN-γ^+^ CD8^+^ T cells and the levels of IFN-γ, and decreasing Foxp3^+^ CD25^+^ CD4^+^ T cells in TME, while increasing serum levels of TNF-α and reducing serum levels of TGF-β [85]. Oral administration of *Bifidobacterium* improves spontaneous antitumor immunity and response to aPD-L1 mAb immunotherapy by increasing CD8^+^ T cells in the TME [86]. The engineered probiotic *E. coli* Nissle 1917 strain has been reported to enhance the antitumor ability and efficacy of immunotherapies by increasing the concentration of L-arginine in TME and the number of tumor-infiltrating T cells that produce TNF [87, 88]. In our study, live El1405 induced extensive remodeling of the TME by up-regulating the levels of TNF-α, INF-γ, and CD8 and down-regulating the levels of CD4, IL-6, IL-10, and TGF-β. The selective immune regulation mediated by El1405HT was manifested as a moderate increase in the levels of TNF-α and CD8, and a selective decrease in the levels of IL-10 and TGF-β. Furthermore, El1405-derived metabolites, including ILA, IAA, L-arginine, and butyrate also elevated CD8 levels within the TME, whereas GABA and El1405CS did not change immune cells and cytokines within the TME. It indicated that live El1405 had the strongest regulatory ability against TME immune factors. Our study suggests that the live El1405 collaboratively participates in anti-tumor immunity by inducing the production of indole derivative metabolites and bacterial structural components (such as peptidoglycan and membrane proteins). However, its potential mechanism of action awaits further study.

Microorganisms in the TME affect cancer progression and treatment effectiveness [89–91]. A recent study detected relatively abundant members of putative oncogenic microorganisms in CRC tumor tissues, including *F. nucleatum*, *E. coli*, *B. fragilis*, *E. faecalis*, *Streptococcus gallolyticus,* and *Peptostreptococcus* spp. [92]. Similarly, we found that *Enterobacter*, *Pseudomonas*, and *Staphylococcus* exhibited higher relative abundances in the PBS group compared to the El1405 treatment group. *Staphylococcus xylosus*, *Lactobacillus animalis,* and *Streptococcus cuniculi* isolated from breast cancer tumors have been found to promote metastatic colonization of tumor cells [93]. This study found that the abundance of *S. succinus* isolated from the tumors in the PBS group was relatively high. Furthermore, oral administration of *S. succinus* increased the proportion of abdominal metastasis of tumors and the expression of VEGF in tumor tissues, indicating that *S. succinus* supplementation increased the vascular permeability of tumors, thereby enabling cancer cells to spread to distant organs through the blood. Little is known about the pathogenic properties of *S. succinus*. *S. succinus* has been isolated from foods, environment, and occasionally human clinical materials [45, 94]. *S. succinus* has also been isolated from melanoma samples and can invade melanoma cells [95]. Our observation of the cancer-inducing properties of *S. succinus* is intriguing, which warrants further investigation. In addition, *S. succinus* significantly enriched potential pathogenic species such as *C. difficile* and *M. sciuri*. *C. difficile* is a well-known intestinal pathogen that can promote colonic tumorigenesis in mice [96]. In this study, in the CT26 syngeneic mouse PBS group, *Staphylococcus* was enriched in the gut and TME, suggesting that it may spread from the gut to the TME through tumor-induced vascular permeability.

We verified that El1405CS could not inhibit the proliferation of human non-small cell lung cancer cell line A549 and the human cervical carcinoma cell line HeLa, whereas El1405 could inhibit the proliferation of digestive cancer cell lines including gastric cancer cell lines AGS and MGC-803 (Fig. S2I). Therefore, we speculate that El1405 mainly inhibits cancers associated with the digestive tract, but further experiments are necessary to confirm this hypothesis. Although chemotherapy is a common treatment method for CRC, drug resistance remains a significant challenge, leading to treatment failure, reduced progression-free survival, and increased cancer recurrence rates [97]. Combining El1405 with antitumor agents, such as cisplatin, 5-FU, paclitaxel, and oxaliplatin, significantly enhances their anti-proliferative effects on CT26 cells (Fig. S2J). This synergistic interaction indicates that El1405 may improve the sensitivity of chemotherapy drugs and potentiate the efficacy of traditional antitumor therapies.

In conclusion, our study demonstrates that *E. limosum* exhibits a significant anti-CRC effect. This effect is associated with the modulation of gut microbiota composition, the remodeling of immunosuppressive TME, and the secretion of antitumor metabolites. These findings may contribute to the development of therapeutic strategies utilizing probiotics for the prevention of CRC.

### Limitations of the study

The study has some limitations regarding mechanism investigation. We have only confirmed the antitumor effect of El1405 in the CT26 syngeneic mouse model. We will need to validate the antitumor effect of El1405 in *Apc*^Min/+^ C57B/6 mice which serves as a model for spontaneous CRC and accurately recapitulates human familial adenomatous polyposis. In addition, we need to use a germ-free mouse model to determine whether the gut microbiota is necessary for El1405 to exhibit antitumor properties. Moreover, we will need to further explore how metabolites protect against CRC.

## Supplementary Material

Supplemental_material_wraf130

## Data Availability

The article and Supplementary Material include the original contributions presented in this study. Further inquiries can be directed to the corresponding author. The GenBank accession number for the whole-genome sequence of strain El1405 is PRJNA1170505. The 16S rRNA gene sequence data from this study are publicly available in the NCBI Sequence Read Archive (SRA) database (Bioproject number: PRJNA1172170 and PRJNA1171310).
