# Supplementary material for: Eubacterium limosum modulates tumor microenvironments and produces antitumor metabolites active against colorectal cancer
Source: ISME J. 2025 Jun 26;19(1):wraf130. doi: 10.1093/ismejo/wraf130 (PMC12477602; doi:10.1093/ismejo/wraf130)
Supplement: Supplemental_material_wraf130 [file supplemental_material_wraf130.pdf]

Supplementary information for

Eubacterium limosum modulates tumor microenvironments and produces antitumor metabolites active against colorectal cancer

Yao Lu[1], Ruiting Lan[2], Qianhua Fan[1, 3], Huijing Tang[1, 3], Dalong Hu[2], Shuwei Zhang[1, 3], Xiaoying Lin[1, 3], Ruoshi Wang[1], Ruiqing Zhao[1], Hui Sun[1], Liyun Liu[1, 4, 5 *], Jianguo Xu[1, 3, 4 *]

*Corresponding authors. Liyun Liu & Jianguo Xu, National Institute for Communicable Disease Control and Prevention, Chinese Center for Disease Control and Prevention, 155 Changbai Road, Beijing, 102206, China. E-mail: liuliyun@icdc.cn; xujianguo@icdc.cn

This PDF includes:

(1) Figures S1 to S3

(2) Table S1 to S4

(3) Supplementary Materials and Methods

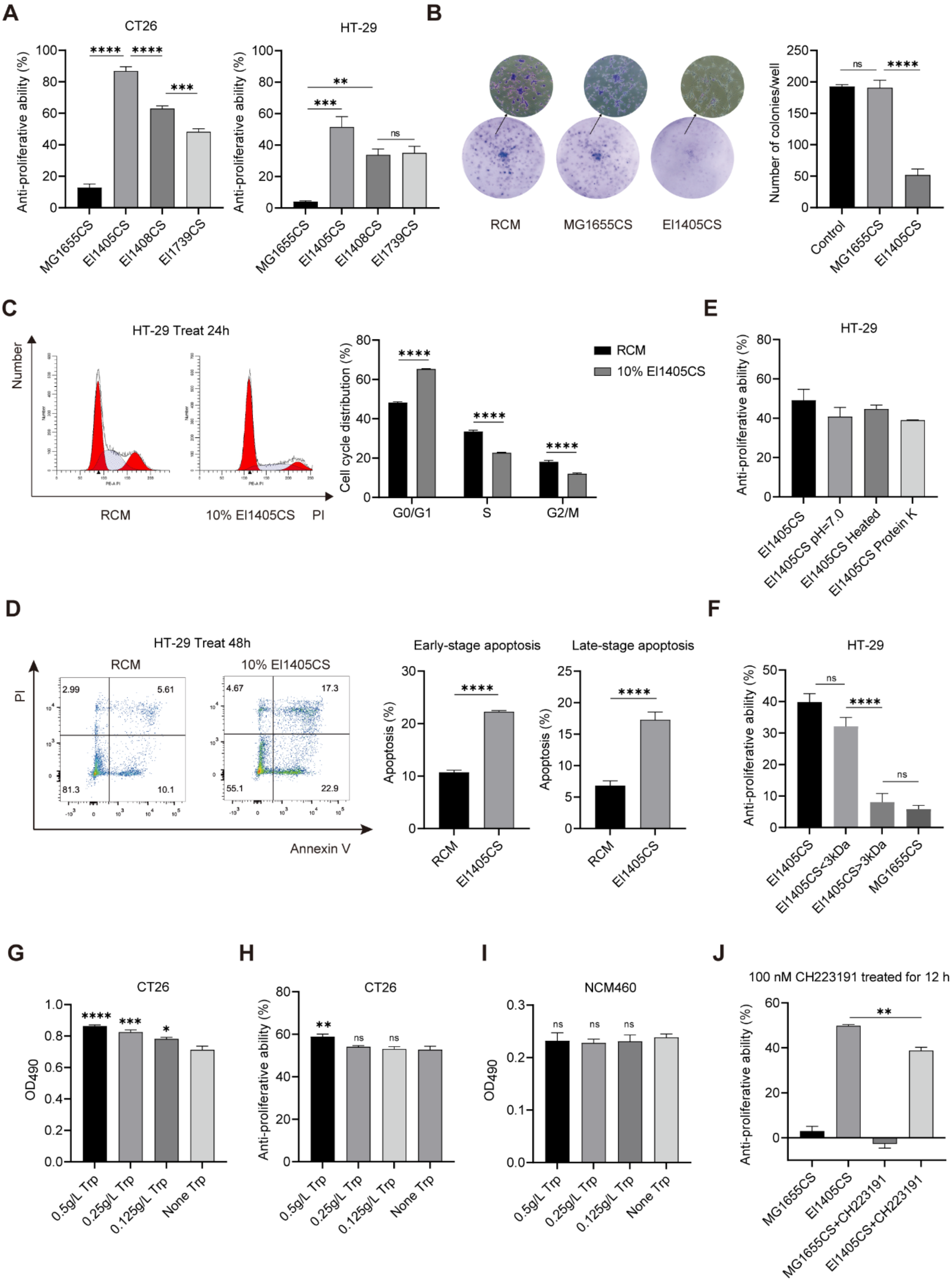

A
CT26
HT-29
Anti-proliferative ability (%)
MG1655CS
EI1405CS
EI1408CS
EI1739CS
B
RCM
MG1655CS
EI1405CS
Number of colonies/well
Control
C
HT-29 Treat 24h
Number
RCM
10% EI1405CS
PI
Cell cycle distribution (%)
G0/G1
S
G2/M
D
HT-29 Treat 48h
Annexin V
Early-stage apoptosis
Late-stage apoptosis
Apoptosis (%)
E
EI1405CS pH=7.0
EI1405CS Heated
EI1405CS Protein K
F
EI1405CS<3kDa
EI1405CS>3kDa
G
OD490
0.5g/L Trp
0.25g/L Trp
0.125g/L Trp
None Trp
H
I
NCM460
J
100 nM CH223191 treated for 12 h
MG1655CS+CH223191
EI1405CS+CH223191

Figure S1. Supplementary results of CRC cells. (A) The anti-proliferative activities of three isolated *E. limosum* strains in CRC cell lines; (B) 10% El1405CS suppressed colony formation of CT26 cells; (C) After 24 h treatment with 10% El1405CS, it induced HT-29 cell cycle arrest at G0/G1 phase; (D) The cell apoptosis of HT-29 cells was significantly increased by El1405CS (10%) at 48 hours; (E) The El1405CS still exhibited inhibitory effects on the proliferation of HT-29 cells after various conditions of treatment; (F) El1405CS < 3 kDa fraction reduced the HT-29 cell viability; (G) Different concentrations of tryptophan were added to the RCM medium, and the medium promoted CT26 cell proliferation as the tryptophan concentration increased; (H) Different concentrations of tryptophan were added to the RCM medium, and with the increase of tryptophan concentration, the ability of cultured El1405CS to inhibit CT26 proliferation was increased; (I) Tryptophan did not affect the proliferation of normal colonic epithelial cells NCM460; (J) Pretreat CT26 cell with AhR-specific antagonist (CH223191) for 12 hours, CH223191 partially abolished the anti-cell-proliferative effect of El1405CS; Statistical significance was determined by one-way ANOVA test with Tukey's multiple comparisons test (A, B, F and J), Dunnett's multiple comparisons test (E, G, H and I), and unpaired two-tailed Student t-test (C and D). Data are presented as mean ± SEM. ns, not significant; *, $p < 0.05$; **, $p < 0.01$; ***, $p < 0.001$, ****$p < 0.0001$. Trp, tryptophan; RCM, Reinforced Clostridium Medium; MG1655CS, *Escherichia coli* MG1655 culture supernatant; El1405CS, *Eubacterium limosum* El1405 culture supernatant.

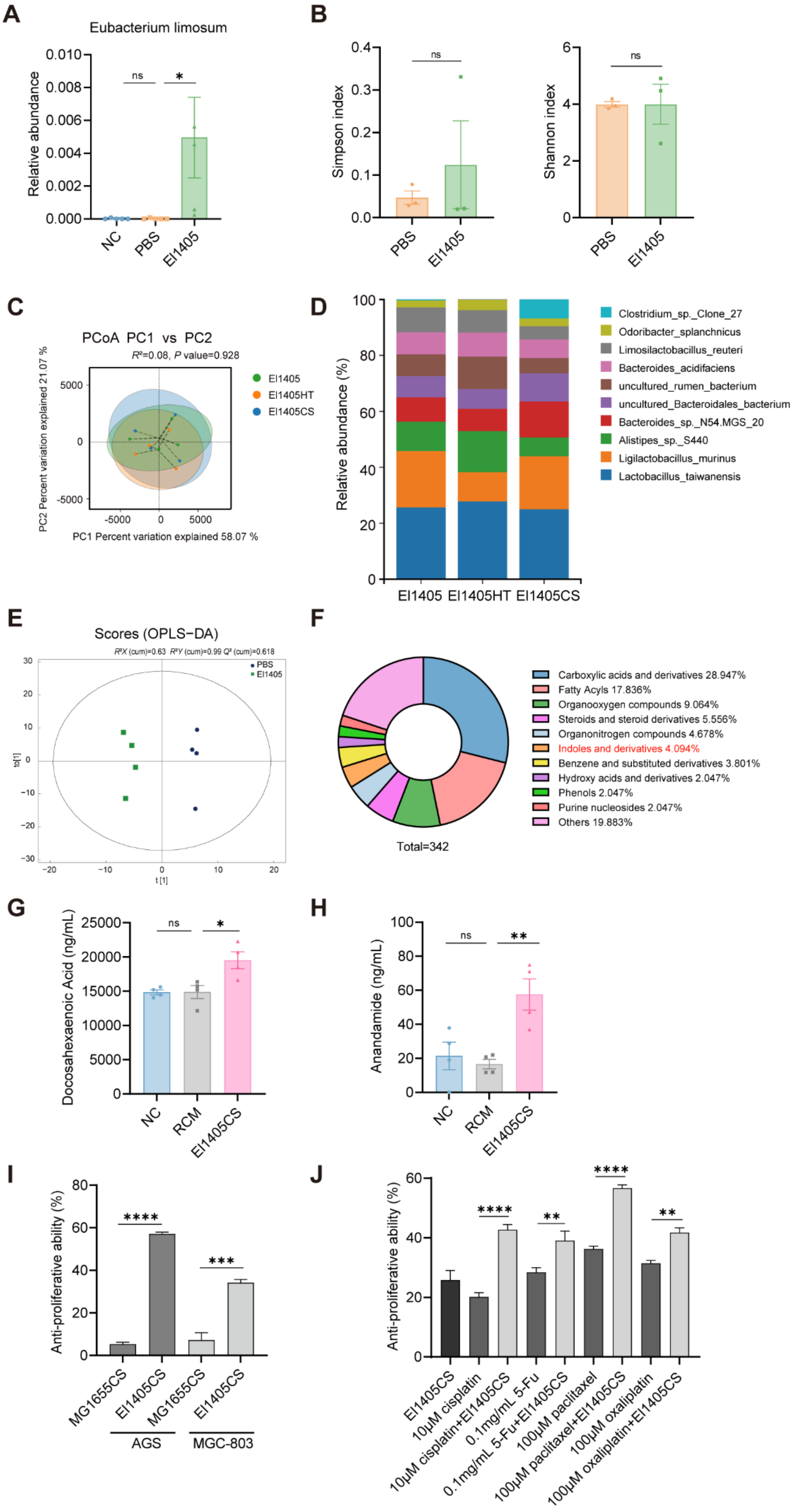
A
Eubacterium limosum
Relative abundance
ns
*
NC
PBS
EI1405
B
Simpson index
Shannon index
ns
PBS
EI1405
C
PCoA PC1 vs PC2
R²=0.08, P value=0.928
EI1405
EI1405HT
EI1405CS
PC2 Percent variation explained 21.07 %
PC1 Percent variation explained 58.07 %
D
Relative abundance (%)
EI1405 EI1405HT EI1405CS
Clostridium_sp._Clone_27
Odoribacter_splanchnicus
Limosilactobacillus_reuteri
Bacteroides_acidifaciens
uncultured_rumen_bacterium
uncultured_Bacteroidales_bacterium
Bacteroides_sp._N54.MGS_20
Alistipes_sp._S440
Ligilactobacillus_murinus
Lactobacillus_taiwanensis
E
Scores (OPLS−DA)
R²X (cum)=0.63 R²Y (cum)=0.99 Q² (cum)=0.618
PBS
EI1405
to[1]
t [1]
F
Carboxylic acids and derivatives 28.947%
Fatty Acyls 17.836%
Organooxygen compounds 9.064%
Steroids and steroid derivatives 5.556%
Organonitrogen compounds 4.678%
Indoles and derivatives 4.094%
Benzene and substituted derivatives 3.801%
Hydroxy acids and derivatives 2.047%
Phenols 2.047%
Purine nucleosides 2.047%
Others 19.883%
Total=342
G
Docosahexaenoic Acid (ng/mL)
ns
*
NC
RCM
EI1405CS
H
Anandamide (ng/mL)
ns
**
NC
RCM
EI1405CS
I
Anti-proliferative ability (%)
****
***
MG1655CS
EI1405CS
MG1655CS
EI1405CS
AGS
MGC-803
J
Anti-proliferative ability (%)
****
**
****
**
EI1405CS
10μM cisplatin
10μM cisplatin+EI1405CS
0.1mg/mL 5-Fu
0.1mg/mL 5-Fu+EI1405CS
100μM paclitaxel
100μM paclitaxel+EI1405CS
100μM oxaliplatin
100μM oxaliplatin+EI1405CS

Figure S2. Supplementary results of CRC cells and animal experiments; (A) In the intestinal contents of the El1405 group, the content of *E. limosum* was significantly increased; (B) Bar graphs of the alpha diversity index between 2 groups. This is shown by the Shannon and Simpson indices; (C) PCoA plots based on Euclidean distance; (D) Barplot analysis of microbiota composition profiling at the species level (top 10); (E) The Orthogonal Partial Least Squares-Discriminant Analysis (OPLS-DA) plot illustrated the differences between the PBS and El1405 groups; (F) All metabolites identified through targeted metabolism were classified and quantified based on their chemical taxonomy, depicted in a ring chart; (G) Docosahexaenoic Acid enriched in serum samples of El1405CS-treated mice (n=4); (H) Anandamide enriched in serum samples of El1405CS-treated mice (n=4); (I) El1405CS (10%) significantly suppressed the cell proliferation of AGS and MGC-803, and MG1655CS was used as a control; (J) The combination treatment of El1405 and antitumor agents significantly enhanced the inhibitory effect of antitumor agents on the proliferation of CT26 cells; Statistical significance was determined by one-way ANOVA test with Tukey's multiple comparisons test (H), and unpaired two-tailed Student t-test (A, B, and G). Data are presented as mean ± SEM. ns, not significant; *, $p < 0.05$; **, $p < 0.01$; ***, $p < 0.001$, ****$p < 0.0001$.

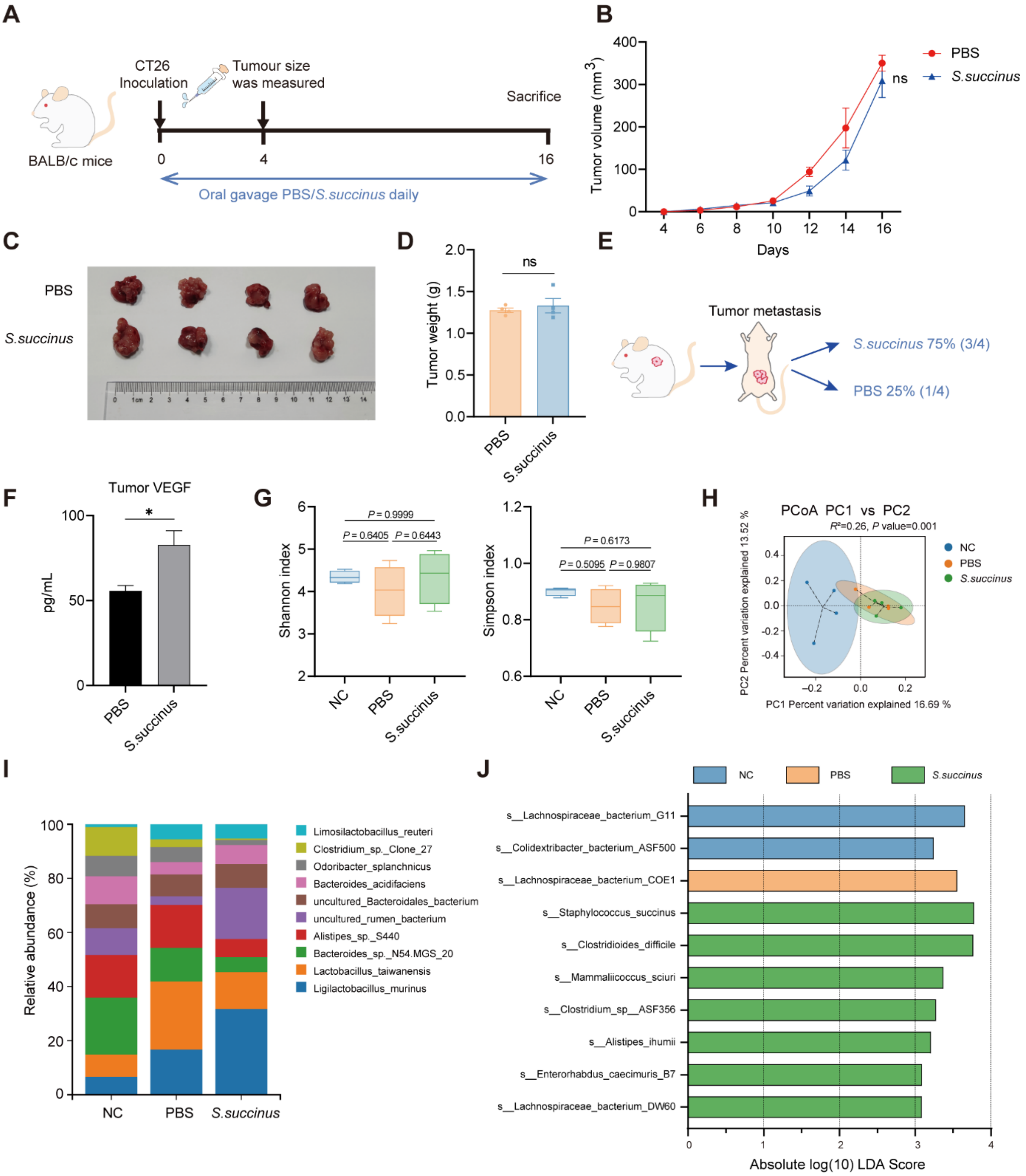
A
CT26
Inoculation
Tumour size
was measured
Sacrifice
BALB/c mice
0
4
16
Oral gavage PBS/S.succinus daily
B
Tumor volume (mm 3)
Days
PBS
S.succinus
ns
C
PBS
S.succinus
D
Tumor weight (g)
ns
PBS
S.succinus
E
Tumor metastasis
S.succinus 75% (3/4)
PBS 25% (1/4)
F
Tumor VEGF
pg/mL
*
PBS
S.succinus
G
Shannon index
Simpson index
P = 0.9999
P = 0.6405
P = 0.6443
P = 0.6173
P = 0.5095
P = 0.9807
NC
PBS
S.succinus
H
PCoA PC1 vs PC2
R²=0.26, P value=0.001
PC2 Percent variation explained 13.52 %
PC1 Percent variation explained 16.69 %
NC
PBS
S.succinus
I
Relative abundance (%)
Limosilactobacillus_reuteri
Clostridium_sp._Clone_27
Odoribacter_splanchnicus
Bacteroides_acidifaciens
uncultured_Bacteroidales_bacterium
uncultured_rumen_bacterium
Alistipes_sp._S440
Bacteroides_sp._N54.MGS_20
Lactobacillus_taiwanensis
Ligilactobacillus_murinus
NC
PBS
S.succinus
J
NC
PBS
S.succinus
s__Lachnospiraceae_bacterium_G11
s__Colidextribacter_bacterium_ASF500
s__Lachnospiraceae_bacterium_COE1
s__Staphylococcus_succinus
s__Clostridioides_difficile
s__Mammaliicoccus_sciuri
s__Clostridium_sp__ASF356
s__Alistipes_ihumii
s__Enterorhabdus_caecimuris_B7
s__Lachnospiraceae_bacterium_DW60
Absolute log(10) LDA Score

Figure S3. *S. succinus* promotes abdominal metastasis of the tumor in the CT26 syngeneic mouse model; (A) Schematic diagram of the CT26 syngeneic mouse model and *S. succinus* gavage schedule; (B) Oral gavage *S. succinus* daily did not influence tumor growth, as evidenced by tumor volume; (C) representative tumor picture, and; (D) tumor weight in the CT26 syngeneic mouse model (n=4); (E) Schematic diagram of tumor metastases in the abdominal cavity after dissection of mice; (F) ELISA for VEGF in the tumor tissue; (G) Boxplots of alpha diversity index among 3 groups. This was shown by the Shannon and Simpson indices; (H) PCoA plots based on binary Jaccard distance; (I) Barplot analysis of microbiota composition profiling at the species level (top 10); (J) Overrepresented bacterial taxa among groups determined by LDA score with a threshold of 3; The statistical significance of the tumor volume was determined by two-way ANOVA test. Statistical significance was determined by one-way ANOVA with Tukey's multiple comparisons test (D and F). Data are presented as mean ± SEM. ns, not significant; *, $p < 0.05$; **, $p < 0.01$; ***, $p < 0.001$, ****$p < 0.0001$.

Supplementary Tables

Table S1. LC-MS/MS quantitative analysis of tryptophan metabolites in EICS1405

abbreviation	Compounds	Molecular Weight	RCM (ng/mL)	EICS1405 (ng/mL)
2-AF	2-Aminophenol	109.05	N/A	774.88
2-AA	2-Aminobenzoic acid	137.05	235.7292	334.76
3-MeOAA	2-Amino-3-methoxybenzoic acid	167.06	N/A	N/A
3-HAA	3-Hydroxyanthranilic acid	153.04	4.14636	7.40
IGA	3-Indoleglyoxylic acid	189.04	N/A	N/A
IAM	Indole-3-acetamide	174.08	N/A	34.56
6-HMLT	6-Hydroxy melatonin	248.12	N/A	N/A
5_HTOL	5-Hydroxytryptophol	177.08	N/A	N/A
5-Me-IAA	5-Methoxyindoleacetic acid	205.07	N/A	N/A
5-HTP	Hydroxytryptophan	220.08	N/A	N/A
TRM	Tryptamine	160.10	32.3838	25.41
SER	Serotonin	176.10	N/A	N/A
IEt	Tryptophol	161.08	104.883	398.49
MLT	Melatonin	232.12	N/A	N/A
5-HIAA	5-Hydroxyindoleacetic acid	191.06	N/A	N/A
L-TRP	L-tryptophan	204.09	29659.08	35427.90
IPA	Indolylpropionic acid	189.08	17.39538	10.41

**ILA**	**Indole-3-lactic acid**	**205.07**	**63.2964**	**2561.42**
ICAld	Indole-3-carbaldehyde	145.05	29.0574	23.13
**IAA**	**β-Indole-3-acetic acid**	**175.06**	**46.90704**	**82.21**
IA	Indole-3-β-acrylic acid	187.06	6.28812	1.86
NAS	N-acetylserotonin	218.11	N/A	N/A
2-KA	2-Ketoadipic acid	160.04	N/A	7.74
NFK	N-formylkynurenine	236.08	64.0116	73.88
L-KYN	L-kynurenine	208.08	4.120506	6.36
CA	Cinnabarinic acid	300.04	N/A	N/A
PA	Picolinic acid	123.03	517.4898	627.57
N-Acid	Nicotinic acid	123.03	1000.314	288.65
QA	Quinolinic acid	167.02	153.1638	130.36
KYNA	Kynurenic acid	189.04	15.16344	18.57
XA	Xanthurenic acid	205.04	57.25782	65.94

Table S2. LC-MS/MS quantitative analysis of amino acids in EICS1405

abbreviation	Compounds	Molecular Weight	RCM (ng/mL)	EICS1405 (ng/mL)
Gly	Glycine	75.07	8120.00	9046.98
Ala	L-Alanine	89.09	19242.79	13268.00
Apa	Beta-Alanine	89.09	N/A	109.00
**Aba**	**4-Aminobutyric acid**	**103.12**	**7614.07**	**23278.07**
Ser	L-Serine	105.09	15983.96	23375.05
Pro	L-Proline	115.13	6813.27	4440.25
Val	L-Valine	117.15	20170.42	39276.19
Thr	L-Threonine	119.12	19675.48	22082.32
Hyp	L-4-Hydroxyproline	131.13	41459.51	468.65
Asn	L-Asparagine	132.12	39452.64	11897.88
Orn	L-Ornithine	132.16	1265.75	2512.80
Asp	L-Aspartic acid	133.10	744.30	5019.33
Gln	L-Glutamine	146.14	12267.68	3940.54
Lys	L-Lysine	146.19	11834.22	62247.52

Glu	L-Glutamic acid	147.13	7752.85	4192.31
Met	L-Methionine	149.21	17181.11	13526.93
His	L-Histidine	155.15	10460.34	12268.84
Phe	L-Phenylalanine	165.19	32945.39	32145.67
--	1-Methyl-L-histidine	169.18	88.38	44.91
--	3-Methyl-L-histidine	169.18	128.17	20.22
**Arg**	**L-Arginine**	**174.20**	**13168.57**	**53089.29**
Cit	L-Citrulline	175.19	519.71	4018.96
Tyr	L-Tyrosine	181.19	6237.85	9255.04
Trp	L-Tryptophan	204.23	14772.97	10212.19
--	N-Acetyl-L-methionine	191.25	43.24	43.90
Iso	L-Isoleucine	131.17	36253.28	39080.41
--	3-Hydroxy-DL-kynurenine	224.21	N/A	N/A
--	L-5-Hydroxytryptophan	220.22	18.78	N/A
Hip	Hippuric acid	179.17	35.77	13.85
--	N-Acetyl-D-phenylalanine	207.23	20.21	28.84
--	D,L-o-Tyrosine	181.19	4639.71	9241.06
--	Nα-Acetyl-L-glutamine	188.18	19.53	133.65
--	L-Carnosine	226.23	2819.43	N/A

Leu	L-leucine	131.17	24585.6<br>0	N/A
Cys	l-Cysteine	121.16	N/A	N/A
--	L-Theamine	174.20	N/A	N/A
--	Glutathione reduced	307.32	99.80	N/A

Table S3. LC-MS/MS quantitative analysis of short-chain fatty acids in EICS1405

Compounds	Molecular Weight	RCM (μg/mL)	EICS1405 (μg/mL)
Acetic acid	60.05	602.89	819.37
Propionic acid	74.08	0.98	1.90
Isobutyric acid	88.11	0.19	0.23
**Butyric acid**	**88.11**	**0.37**	**300.92**
Isovaleric acid	102.13	0.13	0.15
Valeric acid	102.13	0.10	0.49
Caproic acid	116.16	0.09	0.51

Table S4. LC-MS/MS quantitative analysis of metabolites in mice serum between the PBS and EI1405 group

Name	EI1405 mean±SD	PBS mean±SD	Fold change	pvalue	diff
2-Aminooctanoic acid	7.56±0.34	5.52±0.62	1.37	0.001184	up
2-Furoic acid	146.24±43.06	51.68±11.95	2.83	0.005486	up
3-Hydroxybutyric acid	1148±1094.09	6177.51±3621.43	0.19	0.037577	down
3-Methyl-2-oxobutanoic acid	5707.52±562.76	8741.64±2367.71	0.65	0.046942	down
3-Methylhistamine	26.95±2.46	16.71±2.92	1.61	0.001734	up
4-Guanidinobutanoic acid	49.17±17.75	14.85±10.99	3.31	0.016667	up
4-Hydroxybenzoic acid	226.09±122.8	44.47±41.93	5.08	0.03119	up
4-Hydroxycinnamic acid	326.26±30.14	135.87±40.56	2.40	0.000283	up
4-Trimethylammoniobutanoic acid	172.6±17.91	106.71±8.76	1.62	0.000577	up
4Z,7Z,10Z,13Z,16Z,19Z-Docosahexaenoic Acid (DHA)	20027.87±2600.89	15251.88±2336.77	1.31	0.034099	up
5-Aminolevulinic acid	40.35±2.84	26.58±4.75	1.52	0.002516	up
**5-Hydroxyindole-3-acetic Acid**	**414.56±74.33**	**224.01±119.3**	**1.85**	**0.035042**	**up**
7-Dehydrocholic acid (7-	81.25±11.3	43.36±4.6	1.87	0.000805	up

DHCA)					
AICAR	1031.04±54.54	473.21±224.63	2.18	0.00292	up
Alanine	14767.98±1518.57	8602.43±1176.29	1.72	0.000675	up
Anandamide	53.25±28.79	12.24±5.61	4.35	0.031318	up
Anserine	725.41±115.08	345.79±117.28	2.10	0.003612	up
Arachidic Acid	18501.14±1866.67	14885.87±1140.63	1.24	0.0163	up
Asparagine	2964.28±393.43	1687.62±98.74	1.76	0.000749	up
Aspartic acid	2222.17±485.76	481.65±475.82	4.61	0.00218	up
Aspartylphenylalanine	1.96±0.18	1.59±0.14	1.24	0.018426	up
Beta-Alanine	16764.96±1470.72	9904.61±1320.25	1.69	0.000443	up
Butyrylcarnitine	475.67±56.28	358.54±35.38	1.33	0.012456	up
Carnitine	4407.73±47.81	3157.28±581.39	1.40	0.005165	up
Cholesterol sulfate	1695.52±646.81	760.01±384.69	2.23	0.047405	up
Choline	1720.26±183.14	1261.09±220.69	1.36	0.018549	up
Creatine	13095.23±724.53	9063.16±1365.5	1.44	0.001982	up
Creatinine	1468.15±213.81	1050.1±115.05	1.40	0.013741	up
Deoxyadenosine	9.54±1.87	6.84±1.05	1.39	0.045853	up
Dihydroxyacetone phosphate	606.11±127.33	73.35±58.42	8.26	0.000269	up
Dimethylamine	846.35±69.33	511.59±37.99	1.65	0.000148	up
D-Mannosamine	15.58±2.54	7.94±3.61	1.96	0.01342	up

Docosanoic Acid	20148.47±4798.89	11762.84±3151.55	1.71	0.02659	up
Glutamic acid	16066.38±1275.7	10280.74±3535.51	1.56	0.021702	up
Glutarylcarnitine	41.35±4.8	28.5±4.45	1.45	0.007762	up
Glycerophosphocholine	28312.32±1684.75	20274.58±1797.96	1.40	0.000619	up
Glycine	8759.56±615.25	6192.47±793.07	1.41	0.002189	up
Glycoursodeoxycholic acid (GUDCA)	10.51±1.19	5.2±0.35	2.02	0.000141	up
Glycyl-glycine	76.68±20.29	36.86±13.11	2.08	0.016472	up
Homocysteine	277.46±13.16	244.42±15.86	1.14	0.018444	up
Homoserine	12844.38±2716.15	8563.54±932.84	1.50	0.024598	up
**Indole-3-acetamide**	**0.22±0.08**	**0.1±0.03**	**2.14**	**0.026812**	**up**
**Indole-3-carboxylic acid**	**24.93±4.36**	**12.15±7.79**	**2.05**	**0.028617**	**up**
**Indole-3-lactic Acid**	**1414.39±335.37**	**768.91±259.21**	**1.84**	**0.022639**	**up**
**Indoleacetic acid**	**303.52±53.45**	**215.39±28.37**	**1.41**	**0.026878**	**up**
Isovalerylcarnitine	82.48±8.81	62.82±12.11	1.31	0.039317	up
Lactate	296373±36874.96	210785.6±52936.03	1.41	0.03786	up
Linoleoyl ethanolamide	5.38±1.25	2.98±0.67	1.81	0.014785	up
Maltotriose	2908.98±296.64	1098.56±440.35	2.65	0.000488	up
**Melatonine**	**0.02±0**	**0.02±0**	**1.20**	**0.015223**	**up**

Methionine sulfoxide	499.02±105.72	196.63±68.84	2.54	0.00302	up
N2,N2-Dimethylguanosine	7.47±2.03	4.2±1.58	1.78	0.043706	up
N-Acetylcadaverine	0.75±0.11	0.57±0.05	1.31	0.022484	up
**N-Acetylserotonin**	**3.44±0.29**	**2.23±0.43**	**1.55**	**0.003391**	**up**
Oleoylethanolamide	1.42±0.19	0.87±0.23	1.63	0.010002	up
O-Phosphoethanolamine	2491.54±102.62	1168±498.11	2.13	0.002005	up
Ornithine	11155.54±599.88	5845.84±1393.83	1.91	0.000424	up
Ortho-Hydroxyphenylacetic acid	21.09±1.96	14.3±2.55	1.47	0.00557	up
p-Cresyl sulfate	9.8±0.81	5.24±0.49	1.87	7.05E-05	up
Petroselinic acid	4165.5±504.4	3166.81±155.25	1.32	0.00913	up
Phenol	545.13±84.56	407.55±4.57	1.34	0.017488	up
Phosphorylcholine	427.17±29.06	307.26±36.93	1.39	0.002215	up
Pregnenolone sulfate	0.82±0.12	0.5±0.18	1.64	0.023982	up
Proline	2833.81±660.16	1615.78±222.71	1.75	0.012881	up
Quinoline	1.58±0.22	1.14±0.22	1.39	0.031337	up
Ribonic acid	465.34±56.26	250.59±48.36	1.86	0.001163	up
Sebacic acid	8.39±0.34	7.07±0.99	1.19	0.044729	up
Stearic acid	8862.29±1001.69	6618.52±1065.41	1.34	0.021978	up
Suberic acid	1.21±0.07	1.04±0.06	1.16	0.010599	up

Sucrose	468.02±114.28	105.05±88.52	4.46	0.002399	up
Taurocholic acid (TCA)	441.57±119.52	158.03±108.8	2.79	0.012692	up
Threonine	10645.7±2272.76	7036.24±826.09	1.51	0.024472	up
trans-Ferulic acid	75.12±9.14	16.7±18.39	4.50	0.001271	up
Uracil	211.88±61.79	85.01±29.8	2.49	0.010102	up
Valerylcarnitine	138.06±14.38	100.15±20.81	1.38	0.02408	up
Vanillic acid	40.88±17.64	3.15±3.02	12.97	0.005593	up

Supplementary Materials and Methods

Cell culture

HT-29, CT26, and NCM460 cells were cultured in Roswell Park Memorial Institute (RPMI) 1640 medium (Gibco, USA) supplemented with 10% fetal bovine serum (FBS) (Sijiqing, China). Hela and A549 were cultured in Dulbecco's Modified Eagle Medium (DMEM) and DMEM/Nutrient Mixture F-12 medium (Gibco, USA), respectively, supplemented with 10% FBS. Caco-2 was cultured in RPMI 1640 medium supplemented with 20% FBS. All cell lines were cultured at 37°C,5% CO_2. The cells were passaged using 0.25% trypsin-EDTA (Gibco, USA).

Bacterial isolation and culture of intratumoral microbes

0.2g of mouse tumor tissues obtained through aseptic surgery were add to 500μL of Brain Heart Infusion (BHI). After homogenization, they were evenly spread on BHI-5% sheep blood plates and incubated under anaerobic conditions at 37°C for 48 hours. Individual colonies were selected and subcultured on BHI plates. Subsequently, as described above, species identification was performed through 16S rRNA gene sequencing.

Cell viability assay

Cell viability was determined by 3-(4,5-dimethyl-2-thiazolyl)-2,5-diphenyl-2-H-tetrazolium bromide (MTT) assay. Specifically, the bacterial supernatants of different concentrations were added to the RPMI 1640 medium containing 10% FBS and incubated with the cells in a CO_2 incubator at 37°C for 48 hours. Then, the cell viability was detected by the MTT kit (Solarbio, China). As an aryl hydrocarbon receptor (AhR) inhibitor, CH-223191 (Sigma-Aldrich, 100 nM) was added to the cell culture for 12 hours, followed by treatment with bacterial supernatant. To evaluate the anti-proliferative effects of the metabolites, including IAA and ILA, the control group was

administered the corresponding vehicle. These metabolites were respectively solubilized in water, dimethyl sulfoxide (DMSO), or ethanol, ensuring that the final solvent concentration in the treated cells did not exceed 1%. This threshold was established to mitigate the potential cytotoxic effects of DMSO and ethanol on the cells. The remaining bacterial supernatants, not otherwise specified, were applied to the cells at a concentration of 10% (v/v). Antiproliferative activity was calculated by Anti-proliferative ability (%) = (1-$A_{treatment}$ / $A_{control}$) × 100.

In some experiments, cells were treated with metabolites at concentrations of 10 mM, 1 mM, 100μM, or 10 μM. The metabolites include (1) ILA and IAA (Sigma-Aldrich, dissolved in DMSO) (2) L-Arg, GABA, sodium butyrate, and tryptophan (Sigma-Aldrich, dissolved in water) (3) melatonin, 5-Hydroxyindole-3-acetic Acid, indole-3-acetamide, indole-3-carboxylic acid, and N-Acetylserotonin (Aladdin, dissolved in ethyl alcohol).

Apoptosis assay and cell cycle analysis

Flow cytometry was used to detect apoptosis using the FITC Annexin V /Propidium Iodide (PI) Apoptosis Detection Kit I (BD-Pharmingen). PI/RNase Staining Buffer Solution (BD-Pharmingen) was used to evaluate the cell cycle of samples. Briefly, cells were plated in 6-well plates and cultured in RMPI 1640 supplemented with 2% FBS. After 12 h,10% (v/v) EI1405CS or 10% (v/v) RCM was dissolved into a complete medium to replace the old medium. Treated for 24 h to 48 h, both the supernatant and adherent cells were collected by trypsinization. For apoptosis assay and cell cycle analysis, the cells were diluted into a suspension of 1×10^6 cells with PBS, according to the kit's instructions to stain the cell suspension. All samples were acquired on FACSAria III cell Sortor (BD Bioscience) and analyzed by FlowJo_V10 (BD Bioscience) and Modfit LT 5.0 (Verity Software House).

Histological analysis

For histopathological analysis, tumor tissue samples were fixed in 4% paraformaldehyde, dehydrated, and embedded in paraffin wax, after which they were sectioned using a cryotome. The resulting slices were stained with Ki67 (Servicebio) and TUNEL (Servicebio) to assess subsequent pathological changes. For detailed methods of immunohistochemistry, please refer to the previous study [1].

ELISA

Soluble T cell activity markers (CD4/CD8) and cytokines(TNF-α, IFN-γ, IL-6, IL-10, TGF-β) in tumors were detected by enzyme-linked immunosorbent assay (ELISA) kits (Dogesce Beijing, China). A total of 0.1 g of tumor tissue was weighed and then 1 ml of PBS was added for grinding. Subsequently, ELISA detection was performed.

Whole genome sequencing and analysis

DNA was extracted from EI1405 using DNeasy UltraClean Microbial Kit (Qiagen, USA), and then sent to Majorbio for sequencing. The whole genome of EI1405 was obtained by sequencing with NovaSeq™ X Plus (Illumina, USA) and PacBio Sequel IIe (Pacific Biosciences, USA) sequencing platforms. The Illumina libraries were built using the NEXTFLEX Rapid DNA-Seq Kit (Bioo Scientific, USA) according to the provided instructions, ensuring that the genomic DNA is fragmented into segments of approximately 400 bp, while the PacBio libraries were built using the SMRTbell Prep Kit 3.0 (PacBio, USA), following the specified instructions, and fragment the genomic DNA into segments of approximately 10 kb. Single-molecule real-time (SMRT) sequencing was performed on the PacBio Sequel IIe sequencing platform, utilizing SMRT v3.0 cells. To enhance the accuracy of subsequent assembly, the raw reads from Illumina were significantly trimmed using fastp v0.20.0, resulting in high-quality clean data. Unicycler v0.4.8 [2] was then employed to assemble the quality-controlled Illumina reads in conjunction with the HiFi reads from PacBio. Following this assembly,

the Illumina short sequences were mapped onto the assembled genome using Pilon v1.22 [3] for correction. Prodigal v2.6.3 [4] was utilized to predict the coding sequences within the genome. Additionally, sequence alignment tools such as BLASTP v2.15.0 [5] and Glimmer v3.02 [6] were used to functionally annotate the predicted coding sequences (CDS) with various databases, including nr, Swiss-Prot, GO, COG, and KEGG (latest version as of Sep 25th, 2024). Based on previous studies, enzymes related to bacterial tryptophan, L-Arginine, and butyric acid were identified in EI1405, including TrpB, ArAT, and FldH.

Analysis of the 16S rRNA gene sequences

The PowerSoil DNA Isolation kit (Tiangen Biotech, (Beijing) Co., Ltd, China) was used to extract total microbial genomic DNA from the cecal contents and tumor. The primers for full-length 16S rRNA genes were designed as follows: Forward primer 27F: AGRGTTTGATYNTGGCTCAG /Reverse primer 1492R: TASGGHTACCTTGTTASGACTT. The thermal cycling was 2 min at 95°C followed by 25 cycles of 98°C for 10 s, 55°C for 30 s, and 72°C for 90 s, and a final extension at 72°C for 2 min. Sequencing libraries were generated using the SMRTbell Template Prep Kit (PacBio, USA). The amplicon was purified with AMpure PB beads. The purified amplification product was quantified using a Qubit@ 2.0 Fluorometer (Thermo Scientific) and quality-assessed with the Agilent 2100 Bioanalyzer System (Agilent, USA). Finally, the library was sequenced on a Sequel II sequencer (PacBio, USA), yielding 250 bps paired-end reads [7].

Subsequently, quality inspection was conducted on the formed sequencing library, followed by processing, which included barcode recognition, on the high-quality circular consensus sequencing (CCS) sequences obtained. We utilized VSEARCH (version 2.4.3) to cluster sequences at a 97% similarity threshold, thereby deriving the

operational taxonomic unit (OTU) representative sequences [8]. Subsequently, we employed the BLASTn program from BLAST (version 2.9.0) to align the OTU sequences with the SILVA database (www.arb-silva.de/, version 138.2) for species-level taxonomy annotation. We screened for alignments with an e-value < 0.001 and a sequence similarity > 97%. The Lowest Common Ancestor (LCA) method was applied to determine the final species affiliations. For sequences that could not be accurately annotated using BLAST, we further utilized the classify-sklearn classifier in QIIME2 for supplementary annotation, setting the confidence threshold of the classifier at 0.7 [9]. BMKCloud platform (https://www.biocloud.net) was further used for the bioinformatics analysis of this study. We evaluated Alpha diversity through the Simpson and Shannon index to examine the species richness and diversity of samples. We evaluated beta diversity through PCoA, which is measured by calculating the Euclidean, Jaccard, Bray-Curtis, and weighted/unweighted Unifrac distances. The distance with the most significance in each analysis was selected to be shown in the figures (Euclidean in Fig. 3 and Jaccard in Fig. 5, 6, and 9). The screening criteria for linear discriminant analysis effect size (LEfSe) analysis was over 2.5 or 3. We compared any two groups through the Wilcoxon rank-sum test and corrected multiple comparisons through the Benjamin-Hochberg FDR method. Each group included 4 to 6 mice.

Sequencing of the 16S rRNA genes was performed to investigate the change in intratumoral microbes [10]. The amplification of 5R 16S rRNA increases the coverage and resolution of the detection of bacterial species compared with the widely used V4 or V3-V4 amplification. Using the DNA of tumors as a template, specific primers with barcodes were synthesized and multiplexed PCR amplified according to the specified sequencing regions (five regions of 16S rRNA genes: V2, V3, V5, V6, V8). The primer

sequence information is as follows:

V2: F1-TGGCGAACGGGTGAGTAA/ R1-CCGTGTCTCAGTCCCARTG

V3: F2-ACTCCTACGGGAGGCAGC/ R2-GTATTACCGCGGCTGCTG

V5: F3-GTGTAGCGGTGRAATGCG/ R3-CCCGTCAATTCMTTTGAGTT

V6: F4-GGAGCATGTGGWTTAATTCGA/ R4-CGTTGCGGGACTTAACCC

V8: F5-GGAGGAAGGTGGGGATGAC/ R5-AAGGCCCGGGAACGTATT

The thermal cycling consisted of an initial step at 95°C for 3 minutes, followed by 29 cycles of 95°C for 30 s, 53°C for 30 s, and 72°C for 45 s, and a final extension at 72°C for 10 min. The PCR amplification products were quantified using Synergy HTX (Biotek, USA). Sequencing libraries were generated with the NEXTFLEX Rapid DNA-Seq Kit (Bioo Scientific, Austin, Texas, USA). The library was subsequently sequenced using the NextSeq 2000 platform (Illumina, San Diego, USA). The paired-end reads obtained from Illumina sequencing were sorted according to barcodes, resulting in the raw data for each sample. The results from five short amplified regions sequenced were then integrated using the Short MUltiple Regions Framework to determine the community composition and abundance of microorganisms present in samples [11]. The content and methodology of the analysis remained consistent with the aforementioned approach.

Metabolomic analysis

Untargeted metabolomics: UPLC Acquity I-Class PLUS (Waters) and UPLC Xevo G2-XS QTof (Waters) were used to qualitatively and quantitatively analyze the metabolites of the samples, and the metabolites detected in the negative ion mode and positive ion mode were integrated. Progenesis QI software (Waters), METLIN database (https://metlin.scripps.edu), and BMKCloud platform were used for statistical analyses, including Principal component analysis (PCA), Volcano Plot, Venn diagram, and

pathway analysis. Identification of metabolites was used in the Human Metabolome Database (HMDB) and the Kyoto Encyclopedia of Genes and Genomes (KEGG) database [12]. Compared with the RCM medium or control group, specific metabolites enriched in EI1405CS or the treatment group were determined by variable influence on Fold change (Fc) >1.2 or 2, projection (VIP) >1, and p-value < 0.05.

Targeted metabolomics: 1290 Infinity II series UHPLC System (Agilent Technologies) and 6460 Triple Quadrupole Mass Spectrometer (Agilent Technologies) were used to qualitatively and quantitatively analyze the metabolites of the samples. UHPLC-MRM-MS/MS was used for accurate quantitation of the targeted metabolites in the biological sample. Agilent Mass Hunter Work Station Software (B.08.00, Agilent Technologies) was employed for MRM data acquisition and processing.

References

1. Xu H, Luo H, Zhang J *et al.* Therapeutic potential of *Clostridium butyricum* anticancer effects in colorectal cancer. *Gut Microbes* 2023;**15**:2186114. https://doi.org/10.1080/19490976.2023.2186114
2. Wick RR, Judd LM, Gorrie CL *et al.* Unicycler: Resolving bacterial genome assemblies from short and long sequencing reads. *PLoS Comput Biol* 2017;**13**:e1005595. https://doi.org/10.1371/journal.pcbi.1005595
3. Walker BJ, Abeel T, Shea T *et al.* Pilon: an integrated tool for comprehensive microbial variant detection and genome assembly improvement. *PloS One* 2014;**9**:e112963. https://doi.org/10.1371/journal.pone.0112963
4. Hyatt D, Chen G-L, Locascio PF *et al.* Prodigal: prokaryotic gene recognition and translation initiation site identification. *BMC Bioinformatics* 2010;**11**:119. https://doi.org/10.1186/1471-2105-11-119

5. Balakrishnan R, Christie KR, Costanzo MC *et al.* Fungal BLAST and Model Organism BLASTP Best Hits: new comparison resources at the Saccharomyces Genome Database (SGD). *Nucleic Acids Res* 2005;**33**:D374-377. https://doi.org/10.1093/nar/gki023
6. Delcher AL, Bratke KA, Powers EC *et al.* Identifying bacterial genes and endosymbiont DNA with Glimmer. *Bioinforma Oxf Engl* 2007;**23**:673–679. https://doi.org/10.1093/bioinformatics/btm009
7. Yu J, Liu T, Guo Q *et al.* Disruption of the Intestinal Mucosal Barrier Induced by High Fructose and Restraint Stress Is Regulated by the Intestinal Microbiota and Microbiota Metabolites. *Microbiol Spectr* 2023;**11**:e0469822. https://doi.org/10.1128/spectrum.04698-22
8. Rognes T, Flouri T, Nichols B *et al.* VSEARCH: a versatile open source tool for metagenomics. *PeerJ* 2016;**4**:e2584. https://doi.org/10.7717/peerj.2584
9. Bolyen E, Rideout JR, Dillon MR *et al.* Reproducible, interactive, scalable and extensible microbiome data science using QIIME 2. *Nat Biotechnol* 2019;**37**:852–857. https://doi.org/10.1038/s41587-019-0209-9
10. Nejman D, Livyatan I, Fuks G *et al.* The human tumor microbiome is composed of tumor type–specific intracellular bacteria. *Science* 2020;**368**:973–980. https://doi.org/10.1126/science.aay9189
11. Fuks G, Elgart M, Amir A *et al.* Combining 16S rRNA gene variable regions enables high-resolution microbial community profiling. *Microbiome* 2018;**6**:17. https://doi.org/10.1186/s40168-017-0396-x
12. Xu M, Lan R, Qiao L *et al.* *Bacteroides vulgatus* Ameliorates Lipid Metabolic Disorders and Modulates Gut Microbial Composition in Hyperlipidemic Rats. *Microbiol Spectr* 2023;**11**:e02517-22. https://doi.org/10.1128/spectrum.02517-22
